# Organic Semiconducting Hydrogel with Integrated Microbes and Enzymes for Selective Solar CO_2_ Conversion

**DOI:** 10.1021/jacs.6c00205

**Published:** 2026-03-21

**Authors:** Glenn Quek, Beverly Qian Ling Low, Soleh Anderlini, Xian Wei Chua, Marion I. M. Short, Dongseok Kim, Samuel D. Stranks, Erwin Reisner

**Affiliations:** † Yusuf Hamied Department of Chemistry, 2152 University of Cambridge, Lensfield Road, Cambridge CB2 1EW, United Kingdom; ‡ Department of Chemical Engineering and Biotechnology, 2152 University of Cambridge, Cambridge CB3 0AS, U.K.; § Department of Physics, Cavendish Laboratory, 2152 University of Cambridge, Cambridge CB3 0HE, U.K.

## Abstract

Integrating synthetic light-harvesting materials with biological CO_2_-fixing catalysts offers a promising route to efficient and selective solar-to-chemical conversion under mild conditions. However, progress remains limited by the lack of photocatalytic materials that combine biocompatibility, strong electronic coupling with biocatalysts, high biocatalyst loading capacity, and facile product separation. Here we introduce an organic semiconducting hydrogel synthesized from a rationally designed conjugated polyelectrolyte featuring visible-light absorption, water-processability, and covalent cross-linkability. The resulting macroporous, positively charged hydrogel scaffold immobilizes both microbes and enzymes, promoting intimate abiotic–biotic interactions throughout the three-dimensional hydrogel matrix. This platform supports two distinct modes of sacrificial CO_2_ reduction: mediated electron transfer via photogenerated H_2_ to drive acetate synthesis in the microbe *Clostridium ljungdahlii*, and direct electron transfer from photoexcited polymer domains to the isolated enzyme formate dehydrogenase for formate synthesis. By coupling the molecular programmability of organic semiconductors with the selectivity of biocatalysts, this work establishes a versatile class of soft biohybrid materials for solar fuel production through semiartificial photosynthesis.

## Graphical abstract

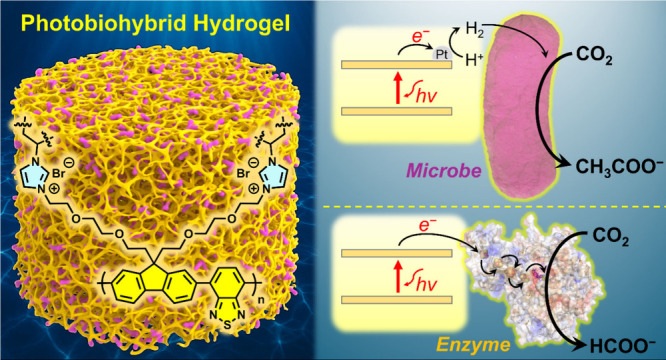

## Introduction

Mitigating CO_2_ emissions and transitioning toward a circular chemical industry are critical for a sustainable future. Solar-powered carbon capture and utilization offer a promising strategy to recycle CO_2_ into fuels and high-value chemicals. Despite the advances in the synthesis of chemicals from CO_**2**_ using sunlight, traditional photocatalytic semiconductors primarily generate simple C1 products from CO_2_ (e.g., carbon monoxide and methane), often with low selectivity, efficiency, and yield.^1–4^ In contrast, nature provides CO_2_-fixing enzymes and bacteria as powerful biocatalysts, offering excellent selectivity and operating efficiently under mild potentials and conditions. Furthermore, leveraging the complex metabolic pathways of bacteria enables the production of more valuable multicarbon products from CO_2_ (e.g., acetate and butyrate).^5–7^ Thus, biohybrid systems that seamlessly integrate the excellent light-harvesting ability of synthetic semiconductors with biocatalysts offer a compelling next-generation approach for solar fuel synthesis.^6–9^

An example of such solar-powered biohybrid systems includes photovoltaic-driven electrolysis and photoelectrochemical (PEC) cells.^10–12^ In many cases, CO_2_-fixing bacteria are coupled to these systems through intermediate electron carriers, such as H_2_. However, the use of diffusible mediators requires either their transport into a separate biocatalyst compartment or operation within an integrated PEC system that suffers from local pH gradients and IR drops.^12,13^ Alternatively, there are enzyme-based systems that can accept electrons directly from (photo)­electrodes.^14,15^ Direct wiring enhances electron transfer and eliminates the need for mediators, but it demands precise control of enzyme orientation and typically relies on surface engineering to ensure robust binding. Despite their relatively high solar conversion efficiencies, solar cells and (photo)­electrodes often require complex fabrication steps, including cleanroom processing, vacuum deposition and lithography.

A more accessible alternative with simpler fabrication involves colloidal systems that employ light-harvesting nanoparticles. These systems enable direct interfacing with biocatalysts, for example through binding near the intraprotein electron transfer chain or active site of an isolated enzyme, or via attachment to the external cell membrane of bacteria.^16–20^ However, many colloidal systems rely on inorganic nanoparticles containing heavy metals (e.g., CdS), which are toxic to living systems and compromise biohybrid stability.^21^ Even enzyme-nanoparticle assemblies utilizing surface functional groups for immobilization often exhibit limited long-term enzyme stability and suboptimal orientation, alongside challenges in product separation and recyclability.^22,23^

Another approach involves immobilizing semiconducting particles into scalable monolithic photocatalyst sheets. These sheets offer a heterogeneous surface for interfacing with microbial or enzymatic biocatalysts, allowing for facile separation of the photocatalyst from reaction products and supporting high performance under biocompatible, near-neutral pH conditions.^24–26^ Nevertheless, these photocatalyst sheets remain limited by several drawbacks, including the extensive use of toxic heavy metals (e.g., Cr_2_O_3_/Ru-SrTiO_3_:La,Rh|ITO|RuO_2_–BiVO_4_:Mo), instability of metal cocatalysts, low surface area for biocatalyst loading, and the requirement for additional interfacial support materials (e.g., ITO on carbon nitride) to ensure strong biocatalyst attachment.^24,26^

A promising yet underexplored strategy involves embedding colloidal photocatalysts within hydrogel scaffolds to enable pseudohomogeneous catalysis. The hydrogel matrices can suppress limitations from nanoparticle aggregation, thereby enhancing stability and preserving surface area for high photocatalytic performance.^27^ Their solid-like nature also facilitates easy recovery and separation from reaction products. Additionally, they offer a tunable porous microenvironment for optimizing mass transport and the incorporation of functional additives such as cocatalysts. Owing to their high water content and intrinsic hydrophilicity, hydrogels also exhibit inherent biocompatibility.^28^ Importantly, their hierarchical porosity can serve as a versatile platform to accommodate biocatalysts across multilength scales — from nanometer-sized enzymes to micrometer-sized microbes — while their tunable surface chemistry promotes robust biocatalyst immobilization. The ease of processing hydrogels into diverse geometries further supports their integration into scalable and adaptable photocatalytic platforms.^29^

To date, most reported biohybrid systems for solar-to-chemical conversion rely on inorganic light-harvesting materials. In contrast, organic semiconductors offer an attractive alternative with synthetically tunable optoelectronic properties, inherent biocompatibility, and a metal-free composition. Nevertheless, their application in whole-cell and enzymatic photocatalysis remains largely underexplored.^22,26,30–33^

Herein, we introduce an organic semiconducting biohybrid hydrogel for solar-driven CO_2_ conversion, based on a newly designed conjugated polyelectrolyte (CPE), **CPE-FBI** — featuring a **f**luorene-*alt*-**b**enzothiadiazole conjugated backbone, functionalized with oligoethylene glycol side chains and vinyl**i**midazolium pendant groups (Figure 1a). This polymer was rationally designed to be water-soluble, absorb visible light, and enable covalent cross-linking. We developed a cryopolymerization method to covalently integrate **CPE-FBI** into a bespoke hydrogel with a macroporous architecture and positively charged surface, thereby facilitating high biocatalyst loading and robust abiotic–biotic interactions within the 3D scaffold. The resulting photoactive hydrogel serves as a versatile platform for integrating either bacteria or enzymes, enabling solar-driven conversion of CO_2_ (Figure 1b,c). This system enables two distinct photobiocatalytic pathways, each selectively yielding a different CO_2_ reduction product: (1) in the mediated electron transfer (MET) mode, photogenerated H_2_ serves as an electron shuttle for CO_2_-to-acetate conversion by the live cell *Clostridium ljungdahlii* (Figure 1b); (2) in the direct electron transfer (DET) mode, the isolated enzyme [W]-formate dehydrogenase (FDH) from *Nitridodesulfovibrio vulgaris* Hildenborough (*Nv*H) receives photoexcited electrons directly from the hydrogel for CO_2_-to-formate conversion (Figure 1c).

**Figure 1:**
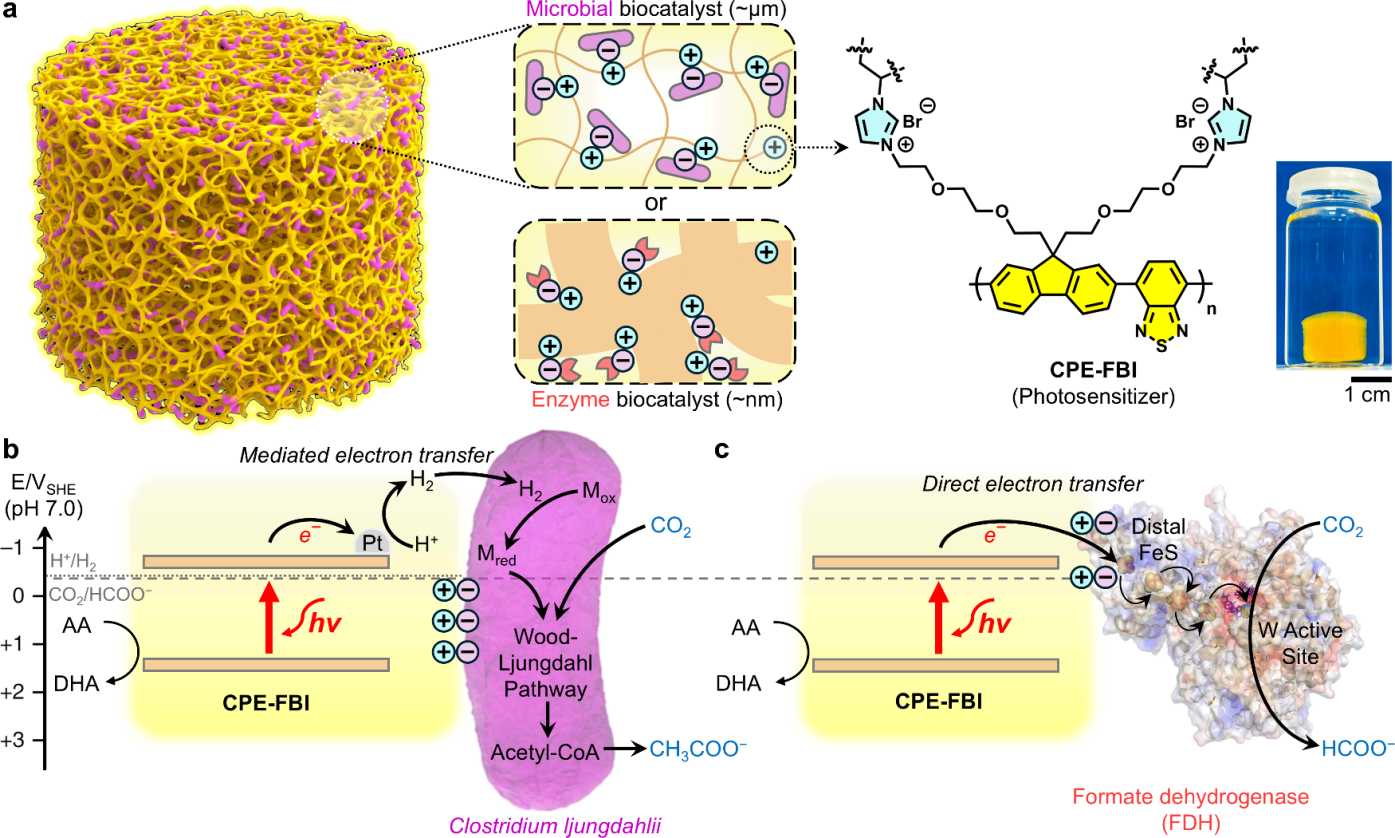
Organic semiconducting biohybrid hydrogels for solar-driven CO_2_ conversion. (a) Concept and molecular design of an organic semiconducting hydrogel integrating microbial and enzymatic biocatalysts. Positive and negative charges illustrate the electrostatic interactions between the cationic **CPE-FBI** and the negatively charged biocatalysts. A photograph of the **CPE-FBI**-containing hydrogel in water (on the top right). Energy level diagram for the (b) photocatalytic production of acetate by a microbial biohybrid hydrogel via mediated electron transfer (MET) and (c) photocatalytic production of formate by an enzymatic biohybrid hydrogel via direct electron transfer (DET). AA = ascorbic acid, DHA = dehydroascorbic acid (both present as sodium salt under experimental conditions).

## Results and Discussion

### Synthesis and Characterization of CPE-FBI

A novel CPE, **CPE-FBI**, was rationally designed and synthesized (Figure 2a). Essential molecular design elements include: 1) a donor–acceptor conjugated backbone of alternating fluorene and benzothiadiazole units, providing a narrow optical bandgap, strong visible-light absorption, and a LUMO energy level thermodynamically suitable for CO_2_ reduction; 2) polar oligoethylene glycol side chains to increase water solubility and hydrophilicity for enhanced photocatalytic H_2_ production; and 3) vinylimidazolium pendant groups that enable covalent cross-linking into hydrogels via free-radical polymerization while promoting electrostatic binding to negatively charged biocatalysts.^34–37^ **CPE-FBI** exhibits excellent aqueous solution processability, low density, intrinsic hydrophilicity, soft properties, and biocompatibility, distinguishing it from typical inorganic photocatalysts.^21,38,39^

**Figure 2:**
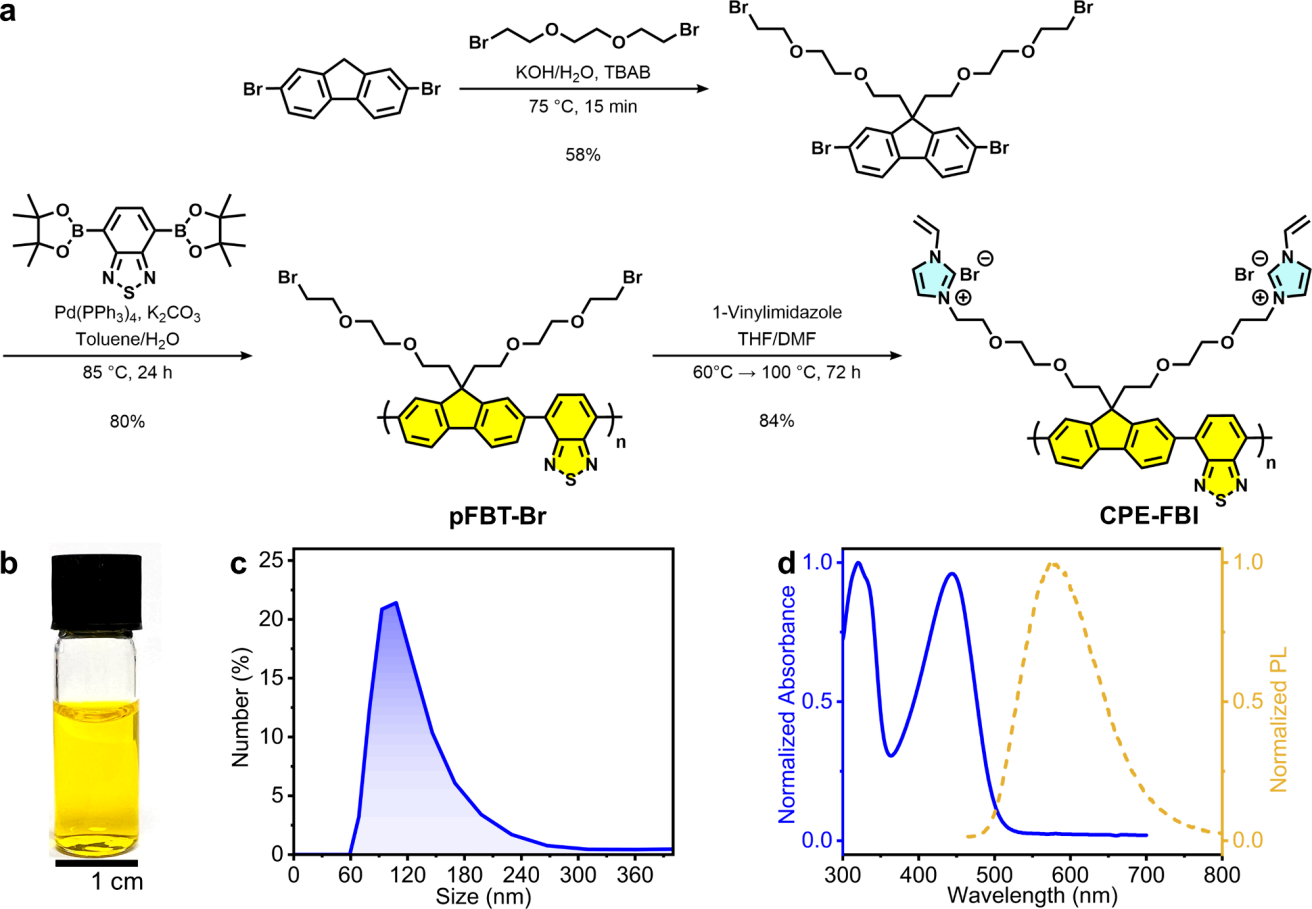
Synthesis and characterization of **CPE-FBI**. (a) Synthetic route for **CPE-FBI**. (b) Photograph of an aqueous solution of **CPE-FBI** (0.5 mg mL^–1^ in water). (c) DLS (dynamic light scattering) size distribution of **CPE-FBI** nanoparticles. (d) Normalized absorbance and PL spectra (λ_exc_ = 450 nm) of **CPE-FBI** in water.

We synthesized **CPE-FBI** via a two-step procedure (Figure 2a; see the Supporting Information for full synthetic procedures and structural characterization, Figures S1–S7). First, the neutral precursor polymer **pFBT-Br** was prepared on a gram-scale via Pd-catalyzed Suzuki coupling in 80% yield. This step was followed by nucleophilic substitution of the bromoalkyl side chains with 1-vinylimidazole to install vinylimidazolium groups on the fluorene units, achieving an 84% yield. The molecular weight of the neutral polymer **pFBT-Br** was determined to be ∼8515 g mol^–1^ (∼12 repeat units of fluorene-*alt*-benzothiadiazole per polymer chain) via gel permeation chromatography (GPC) using chloroform as the solvent (Figure S5). Owing to its polar and ionic side chains, **CPE-FBI** is highly water-soluble and forms stable colloidal nanoparticles in water with diameters of ∼120 nm (Figure 2b, c).

The donor–acceptor-type conjugated backbone of **CPE-FBI** enables strong visible light absorption with a peak at ∼450 nm (ε = 7765 M^–1^ cm^–1^), with a photoluminescence (PL) emission maximum centered at ∼580 nm (Figure 2d). The absorption onset of 585 nm corresponds to an optical band gap (*E*_g_) of 2.12 eV. Electronic energy levels were probed by cyclic voltammetry of an aqueous **CPE-FBI** solution (0.2 mg mL^–1^) using a glassy carbon working electrode and 100 mM tetrabutylammonium bromide (pH 7) supporting electrolyte. The onset reduction potential, corresponding to the LUMO energy level, was determined to be −0.71 V versus SHE (Figure S8). The polymer’s LUMO lies more negative than the reduction potentials for proton reduction to H_2_ and CO_2_ reduction to formate, indicating that **CPE-FBI** provides a thermodynamically favorable driving force as a photocatalyst (Figure 1b, c).

### Synthesis and Characterization of Hydrogels

Covalent incorporation of **CPE-FBI** into a porous hydrogel scaffold was achieved via free-radical polymerization using its vinylimidazolium groups as cross-linking sites. Acrylamide and *N*,*N′*-methylenebis­(acrylamide) were polymerized in the presence of **CPE-FBI** to form a hydrogel, catalyzed by ammonium persulfate and tetramethylethylenediamine (Figure 3a). The gelation process was carried out in a freezer at – 20 °C (i.e., cryopolymerization) to introduce macropores that are templated by ice crystals into the hydrogel for accommodating biocatalysts (Figure 3b).

**Figure 3:**
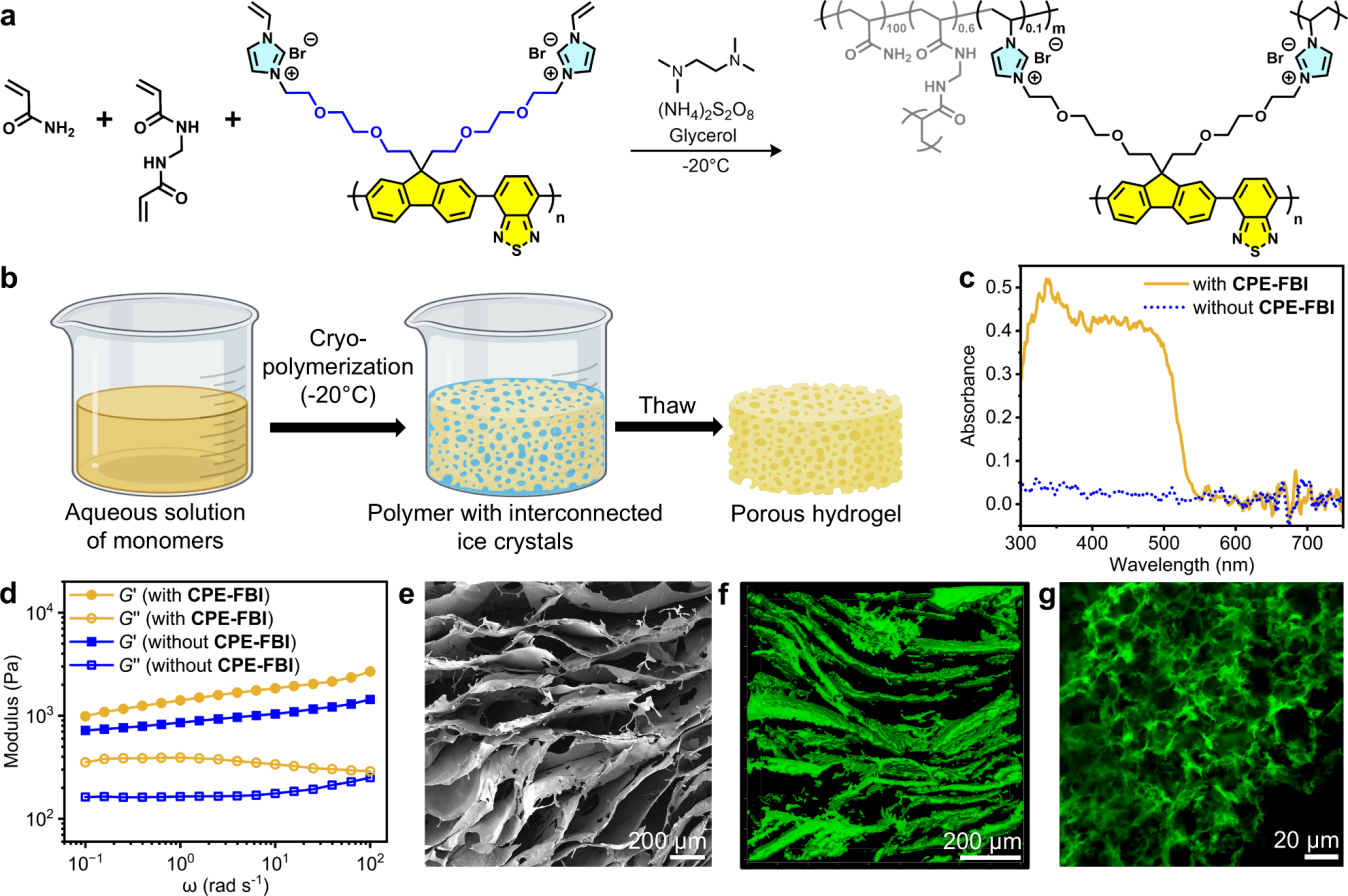
Synthesis and characterization of **CPE-FBI** hydrogels. (a) Radical polymerization of acrylamide, *N*,*N*-methylene bis­(acrylamide) and **CPE-FBI** with ammonium persulfate as the initiator and tetramethylethylenediamine as the catalyst. (b) Pore engineering of the hydrogel via cryopolymerization. (c) Diffuse-reflectance spectra of the hydrogels with and without **CPE-FBI**. (d) Rheological measurements of the elastic (*G*′) and loss moduli (*G*″) as a function of frequency at a constant strain of 0.5% for the hydrogels with and without **CPE-FBI**. (e) Scanning electron microscopy (SEM) image of a freeze-dried hydrogel. (f,g) Confocal laser scanning microscopy (CLSM) images of a hydrogel in its native swollen state in water.

During the initial freezing stage, isolated ice crystals nucleated at multiple points within the pregel solution. As freezing progressed, these ice crystals grew and interconnected, inducing phase separation of the pregel mixture into frozen and nonfrozen regions. The reactive hydrogel components became concentrated in the nonfrozen phase between the ice crystals, where free-radical polymerization proceeded to form the polymer network. Upon completion of polymerization, thawing of the ice crystals yielded a macroporous hydrogel. To enhance the hydrogel’s optical transmittance, glycerol was added to the pregel solution as an antifreezing agent.^40^ Prior to use, the gels were purified to remove residual reagents by immersion in ultrapure water for 72 h under orbital shaking, with daily solvent exchange.

The resulting **CPE-FBI**-containing hydrogel appears yellow and exhibits a diffuse-reflectance spectrum with an absorbance onset of ∼550 nm (Figure 1a, 3c). Its absorbance profile closely matches that of an aqueous solution of **CPE-FBI** (Figure 2d), confirming its successful incorporation into the hydrogel network. Rheological measurements further verified covalent integration, with the **CPE-FBI**-containing hydrogel displaying a higher elastic modulus (*G*′) than the control polyacrylamide hydrogel without **CPE-FBI** (1671 Pa vs 984 Pa) (Figure 3d). This enhancement is attributed to **CPE-FBI** acting as a multifunctional cross-linker, as each fluorene unit contains two reactive vinylimidazolium groups. Quantitative UV–vis absorption analysis of the supernatant after a 72 h leaching period of a freshly synthesized hydrogel indicated a ∼99% covalent cross-linking efficiency of **CPE-FBI** within the polyacrylamide hydrogel network (Figure S9).

To validate the pore-engineering strategy achieved through cryopolymerization, the hydrogel’s internal microstructure and morphology were examined by scanning electron microscopy (SEM) and confocal laser scanning microscopy (CLSM) (Figure 3e-g). SEM images of the freeze-dried hydrogel reveal lamellar structures with macropores (∼200 μm), templated by ice crystals during freezing (Figure 3e).

To visualize the hydrogel in its native swollen state, CLSM was subsequently employed, leveraging the intrinsic fluorescence of **CPE-FBI** and exciting the hydrogel at 430 nm. The z-stack CLSM reconstruction (Figure 3f) shows a 3D microporous lamellar architecture consistent with the SEM observations, along with uniform distribution of emissive **CPE-FBI** throughout the matrix. Higher magnification imaging reveals that the sheet-like structures contain smaller pores of less than 10 μm (Figure 3g). Such hierarchical porosity is anticipated to promote efficient mass transport of substrates and products, while providing pore dimensions suitable for biocatalyst infiltration, encompassing both micrometer-scale microbes and nanometer-scale enzymes.

### Quenching of Photoexcited CPE-FBI by Sodium Ascorbate

We assessed sodium ascorbate as a sacrificial electron donor for **CPE-FBI** hydrogel photocatalysis by probing its ability to reductively quench photogenerated holes. Femtosecond transient absorption (TA) spectroscopy (450 nm excitation, pulse fluence of 6.9 μJ cm^–2^) was performed on hydrogels immersed in anaerobic aqueous solutions, both with and without sodium ascorbate (0.1 M), to measure the differential transmission Δ*T*/*T* of a broadband visible probe upon photoexcitation.

The initial broad positive Δ*T*/*T* signal observed at 500–600 nm overlaps with the steady-state PL spectrum of **CPE-FBI** and is assigned to stimulated emission (Figure 4a,b, Figure S10a,b). Upon addition of sodium ascorbate, its 1/*e* decay time shortens from 47.6 to 4.2 ps, indicating efficient reductive quenching (Figure S10d). These TA spectroscopy findings are further corroborated by time-resolved PL measurements, which show a substantial reduction in the PL lifetime in the presence of sodium ascorbate (Figure S10c). After ∼10 ps, the broad positive Δ*T*/*T* signal inverts to a negative feature (Figure 4b), revealing a photoinduced absorption feature centered at ∼525 nm that broadens to span 500–700 nm and persists beyond the 6.5 ns pump–probe delay window. Notably, this long-lived negative Δ*T*/*T* feature is absent without sodium ascorbate, which could suggest its assignment to energized electrons formed via reductive quenching.

**Figure 4:**
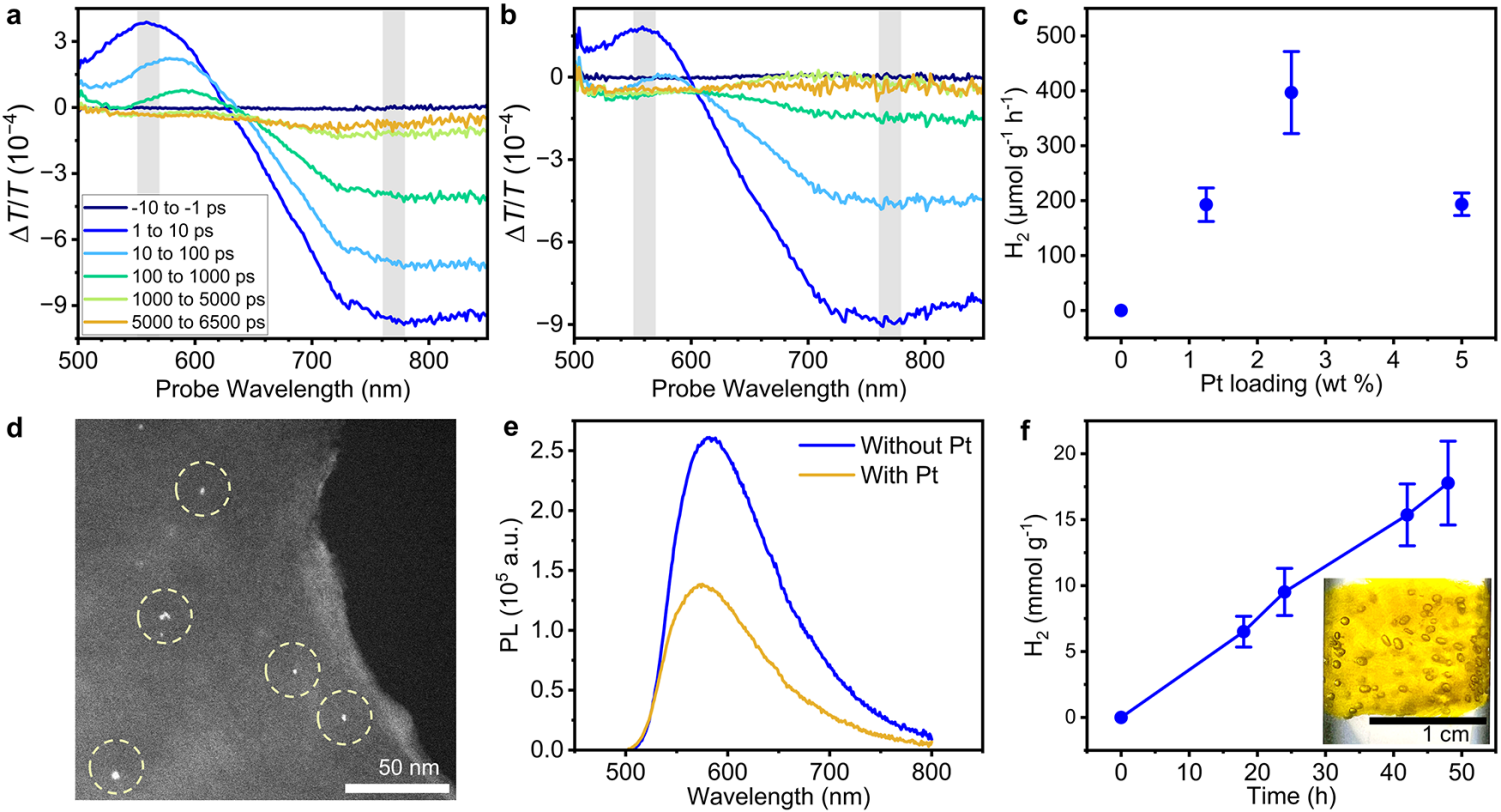
Photocatalytic production of H_2_ by **CPE-FBI** hydrogels. Transient absorption spectra of **CPE-FBI** hydrogels in N_2_-purged aqueous solutions (a) without and (b) with sodium ascorbate (0.1 M), at selected pump–probe time delays (as labeled) up to 6500 ps. The samples are excited at 450 nm (fluence 6.9 μJ cm^–2^). The stimulated emission (550 to 570 nm) and excitonic excited-state absorption features (760 to 780 nm) are highlighted in gray. (c) Photocatalytic H_2_ production by the hydrogels after 24 h of illumination with varying Pt cocatalyst weight loading relative to that of **CPE-FBI**. (d) High-angle annular dark-field scanning transmission electron microscopy (HAADF-STEM) image of the hydrogel after photodeposited with Pt nanoparticles. (e) PL spectra (λ_excitation_ = 450 nm) of the hydrogel in water without sodium ascorbate, before and after Pt photodeposition. (f) Photocatalytic H_2_ production by the hydrogel over 48 h with the optimized Pt loading (2.5 wt%). Inset: photograph of the hydrogel after 48 h showing photocatalytic generation of H_2_ bubbles.

The negative Δ*T*/*T* signal at 760–780 nm (Figure S10e) rises within ∼400 fs and decays over a few nanoseconds, appearing even without sodium ascorbate. This feature could reflect excitonic excited-state absorption; its faster decay in the presence of sodium ascorbate is consistent with exciton quenching via electron transfer.^41^ The 1/*e* lifetimes shorten from 219.8 to 26.1 ps, corresponding to a reductive quenching rate constant of *k* = 3.37 × 10^10^ s^–1^ (*k* = 1/τ_with ascorbate_ – 1/τ_without ascorbate_). Taken together, reductive quenching by sodium ascorbate induces rapid exciton decay and generates long-lived energized electrons, essential for downstream photoreductive catalysis.

### Photocatalytic Production of H_2_

Pt nanoparticles were photodeposited onto **CPE-FBI** hydrogels to serve as cocatalysts for H_2_ evolution. Hydrogels were immersed in 2 mL of water containing 0.2 M sodium ascorbate and K_2_PtCl_6_ (at concentrations of 1.25–5.0 wt% Pt relative to **CPE-FBI**), purged with N_2_, and irradiated under simulated solar light (AM 1.5G, 100 mW cm^–2^) for 16 h at 37 °C.^42^ Following photodeposition, hydrogels were thoroughly washed to remove residual reagents.

The photocatalytic activity of the resulting hydrogels was evaluated under continuous 24 h illumination (AM 1.5G, 100 mW cm^–2^) in biologically relevant conditions, i.e., 2 mL of *C. ljungdahlii* growth medium containing 40 mM sodium ascorbate at 37 °C. No H_2_ was detected in the absence of Pt or sodium ascorbate, confirming that both the cocatalyst and sacrificial electron donor are essential components and that the medium lacks alternative reductive quenchers. An optimal Pt loading of 2.5 wt% yielded the highest H_2_ evolution rate of 397 ± 75 μmol g^–1^ h^–1^ (normalized to the mass of **CPE-FBI**) over 24 h (Figure 4c, Table S1). This H_2_ production capability is enabled by the rational design of **CPE-FBI**, in which oligoethylene glycol side chains increase the semiconductor’s dielectric constant, promote water uptake at photocatalytic sites, and facilitate intimate contact with Pt nanoparticles, thereby improving charge separation efficiency.^34,35^

For an initial Pt loading of 2.5 wt% relative to **CPE-FBI**, inductively coupled plasma optical emission spectroscopy (ICP-OES) revealed a final Pt content of 1.5 wt%, corresponding to a 60% photodeposition yield. High-angle annular dark-field scanning transmission electron microscopy (HAADF-STEM) imaging confirmed the presence of ∼3 nm Pt nanoparticles distributed across the hydrogel surface (Figure 4d). In addition, fluorescence emission from the hydrogel was further quenched following Pt photodeposition, consistent with efficient charge transfer from the photoexcited **CPE-FBI** to Pt nanoparticles (Figure 4e).

A 48 h stability test under continuous illumination showed a linear increase in H_2_ production with no observable loss of activity (Figure 4f). The efficient and durable solar-driven H_2_ generation under biologically relevant conditions underscores the potential of these hydrogels as photocatalysts for biohybrid systems that exploit H_2_ as an electron carrier.

### Photocatalytic Microbial Biohybrid Hydrogel

As a proof of concept, we constructed a living biohybrid hydrogel incorporating *C. ljungdahlii* as a model microbial biocatalyst.^43,44^ *C. ljungdahlii* possesses a well-characterized Wood–Ljungdahl pathway that enables the use of H_2_ as an electron donor for the reduction of CO_2_ to the multicarbon product acetate (Figure 1b).^43^ First, the interaction between **CPE-FBI** and bacterial cells was characterized using zeta potential measurements and CLSM. *C. ljungdahlii* (OD_600_ = 0.15) was incubated in an aqueous solution of **CPE-FBI** (25 μg mL^–1^) for 30 min at 37 °C. As shown in Figure 5a, the zeta potential of the bacteria shifted positively from −27.5 mV to +9.7 mV after incubation with **CPE-FBI**, which itself has a zeta potential of +26.5 mV. This positive shift supports the electrostatic binding of the cationic polymer to the negatively charged bacterial surface.

**Figure 5:**
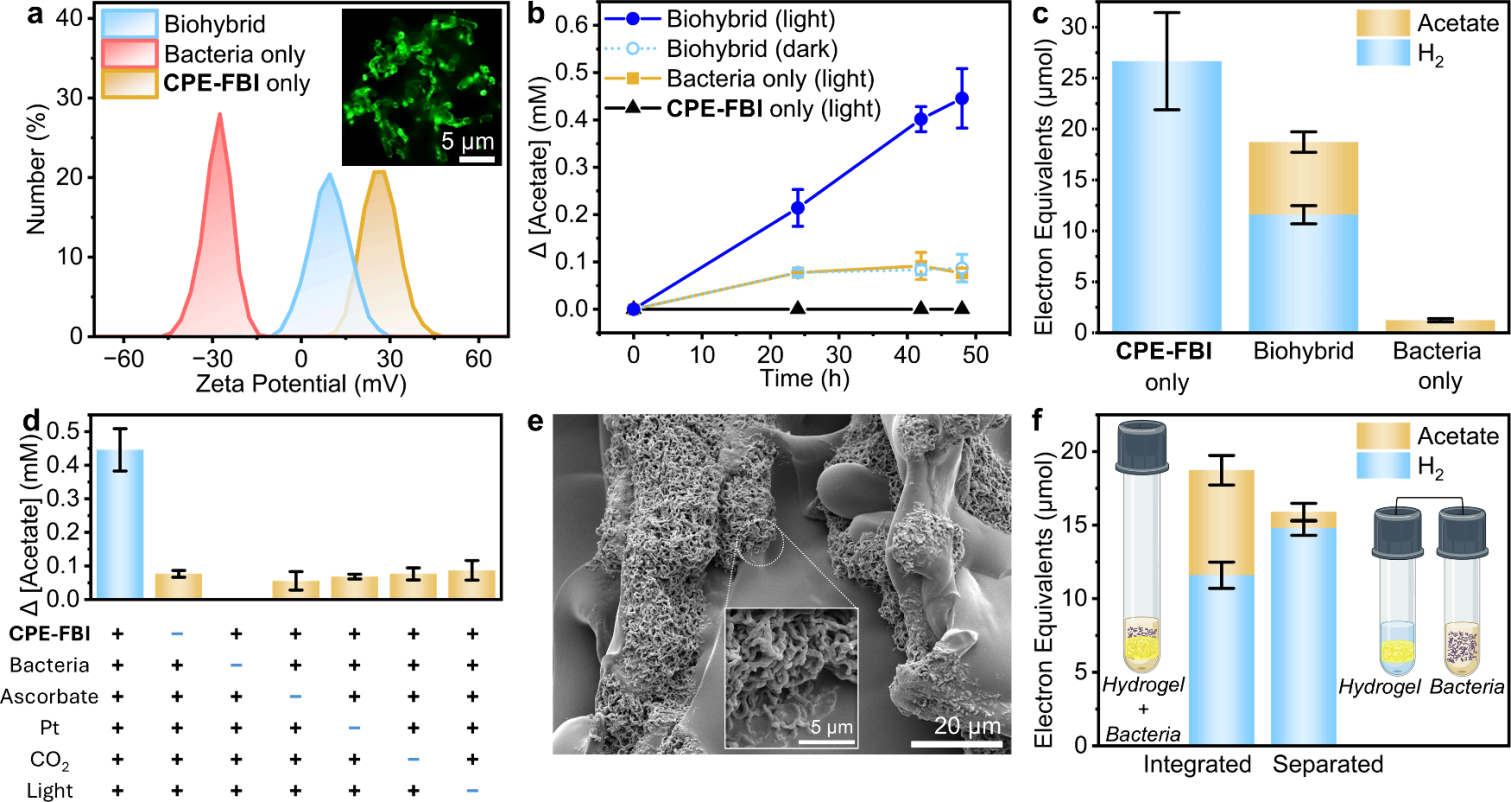
Photocatalytic production of acetate by microbial biohybrid hydrogels. (a) Zeta potential of *C. ljungdahlii* before and after incubating with **CPE-FBI**. Inset: CLSM image of the biohybrid. (b) Acetate production in biohybrids and controls as a function of time. (c) Final product yields after 48 h: H_2_ accumulated in the headspace and acetate produced in the solution. (d) Acetate production after 48 h in exclusion controls. (e) SEM image of the cross-sectioned biohybrid hydrogel, showing extensive colonization of the hydrogel surface by *C. ljungdahlii*. Inset: magnified image of the bacterial cells. (f) Comparison of H_2_ and acetate production between integrated and separated biohybrid configurations after 48 h.

CLSM images further support this interaction, revealing a fluorescent ‘halo’ around the bacterial cells arising from the emissive signature of **CPE-FBI** (inset of Figure 5a). This complementary abiotic–biotic electrostatic interaction is molecularly programmed through the cationic imidazolium groups in **CPE-FBI** and is essential for the effective immobilization of microbial biocatalysts within the hydrogel.

To prepare the biohybrid hydrogel, Pt-photodeposited **CPE-FBI** hydrogels were immersed in a 2 mL *C. ljungdahlii* culture solution (OD_600_ = 0.15), allowing spontaneous infiltration and self-assembly of the negatively charged bacterial cells on the positively charged pore surfaces. The resulting biohybrid was supplemented with sodium ascorbate (40 mM) and purged with 80% N_2_/20% CO_2_ in the headspace. Under 1 sun illumination (AM 1.5G, 100 mW cm^–2^), acetate was successfully produced as the sole detectable product of CO_2_ reduction. The acetate concentration increased almost linearly over 48 h, reaching 0.45 ± 0.07 mM (Figure 5b). In contrast, control samples containing only bacteria produced significantly less acetate (0.08 ± 0.01 mM after 48 h), and production plateaued after 24 h — likely due to the consumption of pre-existing cellular carbon and electron reserves.

No acetate was detected in controls containing only the abiotic hydrogel, confirming that acetate production is biologically driven and not the result of abiotic processes. Furthermore, a control of the biohybrid in the dark showed only minor acetate production (0.09 ± 0.03 mM after 48 h), confirming the light dependence of CO_2_-to-acetate conversion. The apparent quantum yield of acetate production was determined to be 0.3%. These results demonstrate the importance of abiotic–biotic synergy in enabling solar-driven CO_2_ reduction.

The viability of *C. ljungdahlii* was evaluated by monitoring the OD_600_ values of the reaction mixtures (Figure S11). An increase in OD_600_ was observed under photocatalytic conditions for the biohybrid hydrogel over 48 h, whereas no increase was detected in either the dark control or the illuminated bacteria-only control, confirming that bacterial growth was sustained by the photocatalytic activity of the hydrogel. This demonstrates the high biocompatibility and effective functional integration of the organic semiconducting hydrogel with *C. ljungdahlii*. In addition, postcatalytic characterization via PL and FTIR spectroscopies (Figures S12) revealed no significant photodegradation of the **CPE-FBI** hydrogel, indicating robust chemical stability over the 48 h operational period.

Quantification of H_2_ accumulated in the 6 mL headspace after 48 h revealed lower H_2_ levels for the biohybrid hydrogel compared to the abiotic hydrogel (5.8 ± 0.4 μmol vs 13.3 ± 2.3 μmol, Table S2), consistent with H_2_ serving as an electron carrier in the MET process and being actively consumed by *C. ljungdahlii* for the fixation of CO_2_ to acetate. Notably, no detectable H_2_ was observed in the setup containing only bacteria, confirming that H_2_ originates solely from the photocatalytic activity of the hydrogel. This highlights the crucial role of abiotic H_2_ production in driving the biohybrid system, serving as a necessary electron source for microbial CO_2_ fixation.

For a more quantitative electron balance, Figure 5c presents acetate and H_2_ yields converted to electron equivalents, assuming four moles of H_2_ are required to produce one mole of acetate (4*H*_2_ + 2*CO*_2_ → *CH*_3_ *COO* ^–^ + 2*H*_2_ *O* + *H* ^+^), where each H_2_ molecule corresponds to 2 *e* ^–^ and each acetate molecule to 8 *e* ^–^. Based on the difference in H_2_ electron equivalents between the biohybrid setup and the abiotic control, ∼47% of electrons derived from H_2_ consumption by the bacteria were converted to acetate (15.1 ± 3.9 μmol *e* ^–^ from H_2_ consumed versus 7.1 ± 1.0 μmol *e* ^–^ in acetate produced). The remaining electrons were likely directed toward other metabolic pathways and biomass growth.

A series of exclusion control experiments confirmed that the biohybrid system did not produce acetate under solar illumination in the absence of sodium ascorbate, photodeposited Pt, or CO_2_ (Figure 5d, Table S3). The control lacking photodeposited Pt does not produce significant acetate, which shows that CO_2_ fixation by *C. ljungdahlii* occurs primarily via MET using H_2_ as the electron carrier. Additionally, the control without CO_2_ in 100% N_2_ demonstrated that the acetate produced originates primarily from CO_2_.

SEM imaging of cross-sectioned biohybrid hydrogels after 48 h of reaction revealed extensive bacterial infiltration and colonization of the hydrogel surface (Figure 5e), demonstrating the hydrogel’s biocompatibility and its favorable positively charged surface for cell attachment. Furthermore, the proximity of bacterial cells to the Pt-coated photoactive sites creates high local H_2_ concentrations in the microenvironment surrounding the cells, minimizing the diffusion distance of H_2_ for higher overall biocatalytic efficiency. This is supported by a comparison with a separated configuration in which the photoactive hydrogel and bacterial culture were placed in two compartments connected by a tube for H_2_ transfer (Figure 5f). For consistency, the bacteria solution (2 mL) and total headspace (6 mL) were identical to the integrated setup. After 48 h under illumination, the separated system produced only ∼15% of the acetate generated by the integrated biohybrid (1.1 ± 0.6 μmol *e* ^–^ versus 7.1 ± 1.0 μmol *e* ^–^) and consumed less photogenerated H_2_, highlighting the advantage of situating the bacteria directly at the H_2_ evolution sites within the photoactive hydrogel.

Table S4 compares this system with state-of-the-art photocatalytic microbial biohybrids for CO_2_-to-acetate conversion.^16,17,24,30,45^ Direct quantitative comparison is complicated by differences in microbial strains and reaction conditions. A key advantage of our approach lies in its facile fabrication: the biohybrid self-assembles spontaneously upon mixing the hydrogel with bacteria, avoiding the complex biomineralization and washing steps typical of nanoparticle-based systems. In addition, this work introduces a new semiconductor as a soft porous 3D scaffold that contrasts with the conventional solid-state 2D sheets used in previous studies.^24,46^

### Photocatalytic Enzyme Biohybrid Hydrogel

We next investigated the versatility of our photoactive hydrogel by integrating an enzymatic biocatalyst to access alternative CO_2_ reduction products. Having shown that MET via H_2_ is achievable in our microbial biohybrid system, we next sought to establish that DET is also possible in vitro with appropriately selected enzymes, enabled by rationally designed abiotic–biotic electrostatic interactions.

FDH is a well-established enzymatic electrocatalyst for CO_2_-to-formate conversion. While metal-independent FDHs have been coupled to photosensitizers for photocatalytic CO_2_ reduction using redox mediators such as viologens or stoichiometric NAD­(P)­H, some metal-dependent FDHs, including molybdenum- and tungsten-containing FDH (Mo/W-FDH), have been established as reversible, mediator-free CO_2_ reduction catalysts on electrodes and photocatalysts.^47–50^

To this end, we employed wild-type FDH from *Nv*H, which is capable of mediator-free DET for photocatalytic CO_2_ reduction to formate.^22,49^ In this enzyme, electrons are relayed to the buried active site through four iron–sulfur (FeS) clusters, with the outermost (distal) cluster acting as the interfacial electron transfer site. This site is flanked by negatively charged aspartate (Asp) and glutamate (Glu) residues on the protein surface, potentially facilitating interactions with the positively charged **CPE-FBI** and enabling efficient interfacial charge transfer (Figure 1c).^22^

We evaluated the abiotic–biotic interaction using agarose gel electrophoresis. As shown in Figure 6a, the gel illustrates the migration profiles of **CPE-FBI**, FDH, and the biohybrid obtained by incubating **CPE-FBI** with FDH for 30 min. Owing to the intrinsic fluorescence of **CPE-FBI**, its position on the gel is readily visualized under UV illumination, whereas FDH is non-emissive. Fluorescence imaging reveals that **CPE-FBI** alone displays minimal migration under the applied electric field. By contrast, the **CPE-FBI**/FDH biohybrid exhibits a distinct emissive band that has migrated entirely out of the loading well, suggesting comigration of the polymer and FDH and indicative of their association. This interpretation is corroborated by Coomassie blue staining, which shows that the FDH band coincides with the emissive polymer band.

**Figure 6:**
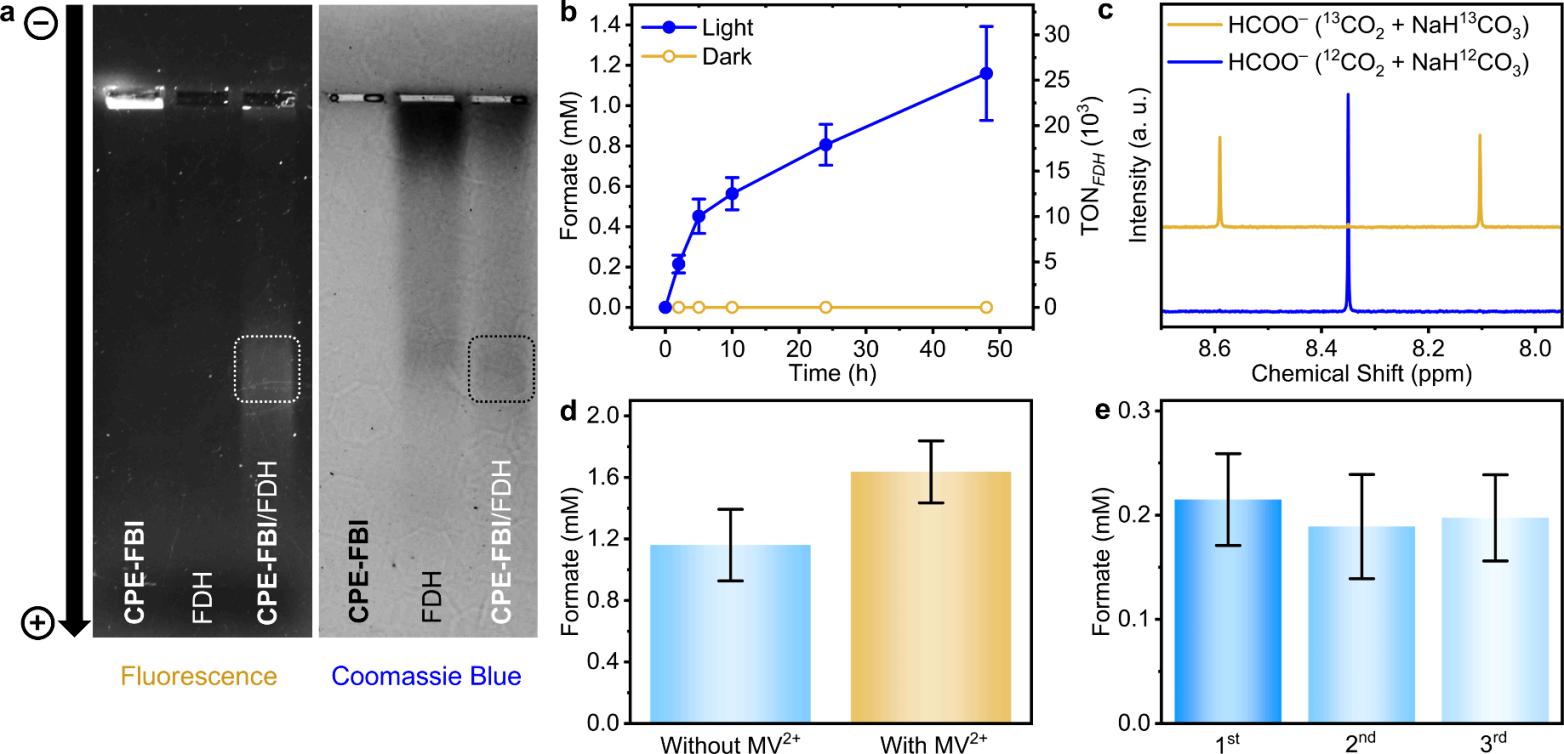
Photocatalytic production of formate by enzyme biohybrid hydrogels. (a) Agarose gel electrophoresis of **CPE-FBI**, FDH, and **CPE-FBI**/FDH biohybrid (left: fluorescence imaging under UV light, right: Coomassie blue protein staining). (b) Time-dependent formate production by the biohybrid hydrogel under light and dark conditions. (c) ^1^H NMR spectra (95:5 H_2_O/D_2_O, 400 MHz) of biohybrid solutions after 48 h of photocatalysis using ^12^CO_2_/NaH^12^CO_3_ (blue line) and ^13^CO_2_/NaH^13^CO_3_ (orange line). (d) Formate production by biohybrid hydrogels with and without MV^2+^ (1 mM) after 48 h of photocatalysis. (e) Formate production over 2 h illumination cycles, with solution exchange between each cycle.

Having established favorable binding between **CPE-FBI** and FDH, pristine **CPE-FBI** hydrogels (without photodeposited Pt) were immersed in an aqueous solution of FDH to form a self-assembled enzymatic biohybrid hydrogel. The solution was a CO_2_-saturated NaHCO_3_ buffer (100 mM, pH 6.7) supplemented with sodium ascorbate (40 mM) as the electron donor. After 48 h of illumination under 1 sun at 30 °C, 1.16 ± 0.23 mM formate was produced, corresponding to a turnover number (TON) of 25748 ± 5169 mol of formate per mole of FDH (Figure 6b). In contrast, no formate was detected in the absence of light, confirming that the process is light-driven.

To confirm that the formate was produced from CO_2_, photocatalytic ^13^C-isotopic labeling was performed using a ^13^CO_2_-saturated electrolyte containing NaH^13^CO_3_ (24 mM). After 48 h of illumination, ^13^C-formate (H^13^COO^–^) was generated as the sole product, as evidenced by a doublet at δ = 8.35 ppm (*J* = 195 Hz) in the ^1^H NMR spectrum, resulting from ^1^H–^13^C coupling (Figure 6c).

The efficiency of photocatalytic DET in the semiartificial hydrogel was probed by introducing methyl viologen (MV^2+^) as a soluble redox mediator to relay electrons to the distal FeS cluster of FDH regardless of proximity to the photoactive sites of **CPE-FBI**.^22,51^ Notably, in the absence of MV^2+^, no exogenous redox mediators were present, meaning formate production under these conditions is attributable exclusively to DET. With the addition of 1 mM MV^2+^, formate production increased only modestly (1.16 ± 0.23 mM to 1.64 ± 0.20 mM after 48 h), indicating that most of the enzyme population is already “electronically wired” to **CPE-FBI** for DET (Figure 6d, Table S5). This high formate_DET_/formate_MET_ ratio of 0.7 indicates that most FDH enzymes are optimally oriented within the hydrogel matrix to enable intimate abiotic–biotic interfacial coupling and that electron transfer between the hydrogel and enzyme is not a limiting step. We attribute this to preferential orientation of FDH via its distal FeS cluster on the soft, positively charged **CPE-FBI** hydrogel surface.

Binding stability of FDH within the hydrogel was assessed through multiple photocatalytic cycles of 2 h each, with the reaction medium fully replaced between cycles using fresh CO_2_-saturated NaHCO_3_ solution (100 mM, pH 6.7) containing sodium ascorbate (40 mM). Formate production remained relatively constant over three cycles, indicating that FDH introduced during the first cycle remains stably retained in the hydrogel to support continuous photocatalytic DET (Figure 6e, Table S6). This observation is consistent with the gel electrophoresis results, which reveal strong electrostatic interactions between **CPE-FBI** and FDH.

The developed enzyme biohybrid hydrogel significantly outperforms previous systems that operate via MET and demonstrates comparable or superior activity to the best performing DET-driven photocatalysts reported to date (Table S7).^22,23,26,47,49,52–56^ More specifically, the hydrogel attains a TOF of 2.0 × 10^3^ h^–1^ (over 5 h) and an apparent quantum yield of 0.3%, while maintaining photocatalytic activity for at least 48 h. Notably, this approach enables direct interfacing of the organic photosensitizer with FDH without requiring additional binding supports (e.g., TiO_2_ and ITO).^26,49^ The hydrogel incorporates a metal-free imidazolium functionality within the polymer network that electrostatically anchors FDH. Moreover, it operates with sustained catalytic activity for up to 48 h, highlighting its robustness and stability.

### Comparison to State-of-the-Art

We present an advance in the design of biohybrid photocatalysts through a modular soft-matter platform capable of integrating both whole-cell and enzymatic biocatalysts. In contrast to conventional colloidal dispersions, which often rely on toxic, heavy-metal-based semiconductors, our approach heterogenizes an organic photosensitizer within a 3D hydrogel matrix. This enables the facile self-assembly of biohybrids through simple diffusive integration, bypassing the complex biomineralization and multistep purification procedures required for nanoparticle-based systems. The solid-like nature of the hydrogel further allows straightforward catalyst recovery and separation from reaction products. Moreover, unlike previously reported solid-state platforms, this hydrogel-based system broadens the materials design space by offering a soft, biocompatible scaffold that accommodates high biocatalyst loading within its pores while providing favorable microenvironments.

More importantly, this work expands the use of underexplored organic semiconductors in constructing photocatalytic biohybrids, a field still largely dominated by inorganic materials. We capitalize on the synthetic tunability of organic semiconducting polymers to molecularly engineer the hydrogel surface for high-affinity electrostatic binding to biocatalysts without the need for additional binding materials, thereby streamlining the abiotic–biotic interface.

This work opens new opportunities for next-generation, metal-free biohybrid systems that are lightweight, biocompatible, and solution-processable, with the versatility to be fabricated into diverse and scalable form factors, including 3D-printed architectures for optimized light capture and floating artificial leaves. Moreover, the hydrogel’s broad compatibility with both whole cells and enzymes holds promise for constructing multibiocatalyst assemblies for the cascade synthesis of complex products.

## Conclusions

We present a first-in-class organic semiconducting biohybrid hydrogel that enables solar-driven CO_2_ conversion into value-added products with tunable selectivity for acetate or formate. The **CPE-FBI** polymer was designed to combine visible-light absorption, water processability, covalent integration into a hydrogel matrix, and a cationic surface for robust biocatalyst immobilization. The resulting photoactive hydrogel exhibits hierarchical porosity and supports high biocatalyst loadings. Its versatility is demonstrated by successful integration with both microbial and enzymatic biocatalysts. Furthermore, the photobiocatalytic pathway can be directed through either MET or DET, depending on the biocatalyst that is modularly integrated. This work provides a new strategy for the design of soft biohybrid materials for semiartificial photosynthesis. Future efforts will focus on eliminating sacrificial electron donors, for example by constructing Z-scheme architectures incorporating complementary photocatalysts for water or waste oxidation, or by developing organic semiconducting polymers with sufficiently deep HOMO energy levels to directly drive valuable oxidative half-reactions.^23,57^ More broadly, molecular engineering of organic semiconducting polymers offers opportunities to integrate additional functionalities, including direct air CO_2_ capture, biodegradability, and expanded solar spectrum utilization.

## Experimental Section

### Materials and Chemicals

All materials and chemicals were purchased from Sigma-Aldrich unless otherwise stated. Reaction gases CO_2_ with 2% CH_4_ as internal gas chromatography standard, and synthesis gas (syngas) cylinders (25% CO, 10% H_2_, 65% CO_2_) were purchased from Brin’s Oxygen Company (BOC). *Clostridium ljungdahlii* DSMZ 13528 was purchased from The Leibniz Institute DSMZ - German Collection of Microorganisms and Cell Cultures GmbH. [W]-FDH from *Nv*H were expressed and purified according to previously reported methods.^58^

### Culturing of *C. ljungdahlii*

*C. ljungdahlii* (internal strain designation RLM034 for strain requests^59^) cultures were grown in ATCC Medium 1754, supplemented with 5.00 g L^–1^ xylose and purged with syngas. After incubation at 37 °C in a shaking incubator for 4 days, an OD_600_ of ∼1.6 was reached. Cells were centrifuged and washed with ATCC Medium 1754 three times, followed by resuspension to a final OD_600_ of ∼0.15 for subsequent experiments.

### Synthesis of Photoactive Hydrogels

10 mL of pregel solution was prepared by mixing ultrapure water (896 μL), 1 mg mL^–1^ **CPE-FBI** (7500 μL), 100% acrylamide (646 μL), 2% *N*,*N*′-methylenebis­(acrylamide) (404 μL), glycerol (494 μL), 10% ammonium persulfate (49 μL), and tetramethylethylenediamine (11 μL). 1 mL of the pregel solution was injected into a rubber mold, followed by cryo-polymerization in a –20 °C freezer for 48 h. The frozen gels were removed from the rubber mold and allowed to thaw at room temperature. The thawed gels were purified to remove residual reagents by immersion in ultrapure water for 72 h under orbital shaking, with the water exchanged every 24 h.

### Photodeposition of Pt Cocatalyst on Hydrogels

Hydrogels were immersed in 2 mL of ultrapure water containing sodium ascorbate (0.2 M) and K_2_PtCl_6_ (with varying Pt loadings of 1.25 wt% to 5 wt% relative to **CPE-FBI**). The solution was purged with 100% N_2_ gas, then exposed to simulated solar irradiation (1 sun, Air Mass 1.5 Global (AM 1.5G) filter, 100 mW cm^–2^) for 16 h to deposit Pt nanoparticles within the hydrogels. After photodeposition, the hydrogels were washed to remove unreacted chemicals before use in subsequent photocatalytic experiments.

### Photocatalytic H_2_ Production of Hydrogels

Hydrogels photodeposited with Pt were immersed in 2 mL of ATCC Medium 1754 containing sodium ascorbate (40 mM). The solution was purged with 98% N_2_/2% CH_4_ gas, then exposed to simulated solar irradiation (1 sun, Air Mass 1.5 Global (AM 1.5G) filter, 100 mW cm^–2^) with a 400 nm long-pass filter at a constant temperature of 37 °C. Gaseous H_2_ in the headspace were analyzed by gas chromatography using a Shimadzu Tracera GC-2010 Plus with a barrier discharge ionization detector, equipped with a ShinCarbon micro–ST Column (0.53 mm diameter) kept at 40 °C using Helium carrier gas. Typically, 50 μL of headspace gas were injected using a gastight syringe (Hamilton). The response factors for the gases were determined by calibration with known amounts of H_2_.

### Photoexperiments for CPE-FBI/*C. ljungdahlii* Biohybrids

Hydrogels photodeposited with 2.5 wt% Pt were immersed in 2 mL of *C. ljungdahlii* culture (OD_600_ = 0.15) containing sodium ascorbate (40 mM). The solution was purged with 80% N_2_/20% CO_2_ gas, then exposed to simulated solar irradiation (1 sun, Air Mass 1.5 Global (AM 1.5G) filter, 100 mW cm^–2^) with a 400 nm long-pass filter at a constant temperature of 37 °C. Acetate production was quantified by quantitative ^1^H nuclear magnetic resonance (qNMR) spectroscopy using a Bruker 400 MHz Neo Prodigy Spectrometry. The chemical shifts (δ) in the ^1^H NMR spectra were referenced to the singlet peak of the internal standard 3-(trimethylsilyl)­propionic-2,2,3,3-d_4_ acid, sodium salt (0.075 wt% in D_2_O).

### Photoexperiments for CPE-FBI/FDH Biohybrids

Pristine hydrogels without Pt were immersed in 2 mL of CO_2_-saturated NaHCO_3_ solution (100 mM, pH 6.7) with sodium ascorbate (40 mM) and FDH (50 pmol), then exposed to simulated solar irradiation (1 sun, Air Mass 1.5 Global (AM 1.5G) filter, 100 mW cm^–2^) with a 400 nm long-pass filter at a constant temperature of 30 °C.

### Apparent Quantum Yield (AQY) Calculations

The apparent quantum yields (AQY) were calculated according to the following equations:

$${AQY}_{acetate}\left(\%\right)=\frac{8\times C_{acetate}\times V\times N_{A}}{\phi_{photo}\times t\times A}\times 100$$

$${AQY}_{formate}\left(\%\right)=\frac{2\times C_{formate}\times V\times N_{A}}{\phi_{photo}\times t\times A}\times 100$$

where *C* is the concentration of product, *V* is the volume of the reaction mixture, *N*_*A*_ represents Avogadro’s Number, ϕ_*photo*_ represents the photo flux, *t* is the irradiation time, and *A* is the area of light irradiation. The light intensity (*P*, mW cm^–2^) from a LOT MSH300 monochromator and a LOT LSH302 light source with a 300 W Xe lamp was measured using a Thorlabs PM100D power meter with a Thorlabs S302C thermal power sensor head, and converted to ϕ_*photo*_ by the following equation:

$$\phi_{photo}=\frac{P\times \lambda }{h\times c}$$

where λ, *h*, and *c* represent the wavelength (450 nm), the Planck constant, and the speed of light in vacuum, respectively.

### Scanning Electron Microscopy (SEM)

After the photoexperiments, the microbial biohybrid hydrogels were washed three times with ATCC Medium 1754 and fixed with 2.5% glutaraldehyde at 4 °C overnight. Then, the glutaraldehyde was removed, and the hydrogels were washed twice with ultrapure water. The hydrogels were washed successively with 10, 30, 50, 70, 90, and 100% ethanol for 6 min each before leaving to dry in air overnight. Dried hydrogels were then sputtered with platinum and imaged with Tescan Clara 2 SEM.

### Transmission Electron Microscopy (TEM)

A thin piece of hydrogel was placed onto a carbon coated 200 mesh copper EM grid and left to dry overnight before imaging on a Thermo Fisher Scientific Talos F200X G2 S/TEM.

### Confocal Laser Scanning Microscopy (CLSM)

The intrinsically fluorescent **CPE-FBI** hydrogel was placed in a Nunc Lab-Tek II Chamber Slide and imaged using a LEICA Stellaris 5 Confocal microscope with an excitation wavelength of 430 nm.

### Rheological Measurements

Measurements were performed using an Anton-Paar MCR302 Rheometer. Experiments were performed in a strain-controlled mode. The hydrogels were deposited onto a plate and a frequency sweep was done at a constant strain of 0.5%.

### Isotopic Labeling

The reaction solution was modified by changing the NaH^12^CO_3_ into NaH^13^CO_3_, and ^13^CO_2_ was used to purge the reaction solution before photocatalytic experiments.

### Agarose Gel Electrophoresis

Agarose gels were prepared by dissolving 0.8 g of agarose in 100 mL of 1× TAE buffer (40 mM Tris, 20 mM acetic acid, and 1 mM EDTA), followed by heating until the agarose was fully dissolved. The resulting 0.8% agarose solution was poured into a gel mold with a well comb in place and allowed to solidify at room temperature for 30 min. Once set, the gel was transferred to an electrophoresis cell filled with 1× TAE buffer. The biohybrid sample was prepared by mixing **CPE-FBI** (7 μg mL^–1^) with FDH (3.3 μM) and incubating the mixture inside a glovebox under a humid argon atmosphere for 30 min. Individual samples of **CPE-FBI**, FDH, and the biohybrid (10 μL) were each mixed with 5 μL of loading buffer (containing 8 mg bromophenol blue, 4 mL glycerol, and 5 mL of 0.5 M Tris, pH 7), then loaded into separate wells of the gel. Electrophoresis was carried out at 150 V for 1 h. The gel was imaged under UV light using a ChemiDoc imager. FDH was visualized using Coomassie Blue staining.

### Picosecond to Nanosecond Transient Absorption Optical Spectroscopy

Measurements were performed on a HARPIA-TA system (Light Conversion). The output of a Yb:KGW laser (PHAROS, 1035 nm, 10 kHz repetition rate, 163 fs pulse width, Light Conversion) was divided into two paths. One beam was directed to an optical parametric amplifier (ORPHEUS-NEO) to generate the 450 nm pump pulse. The second beam was focused onto a 5 mm sapphire crystal to generate a continuum probe in the visible range. The magic angle of 54.7° was set between the polarization of the pump and probe beams. A mechanical delay stage varied the time delays between the pump and the probe beams. The pump and probe beams were spatially overlapped on the sample, and the transmitted probe was collected using an Andor Kymera 193i spectrograph.

### Transient Photoluminescence Spectroscopy

Time-correlated single photon counting (TCSPC) measurements were performed on an Edinburgh Instruments FLS1000 fluorimeter, using a picosecond pulsed diode laser (HPL-450) emitting at 450 nm. The laser was connected to the spectrometer via a coupling flange and operated at a repetition rate of 5 MHz, with an average power of 0.81 mW, corresponding to an energy of 162 pJ per pulse. The detection wavelength was fixed at 580 nm. The instrument response function (IRF) was obtained by detecting the reflection of the excitation beam from an empty cuvette, with the detection wavelength set to the excitation wavelength (450 nm).

### Statistics

All error bars represent the mean ± standard deviation (n = 3 independent samples). The calculation was processed using OriginPro 2024 (OriginLab).

## Supplementary Material



## Data Availability

The raw data underpinning the findings of this study can be accessed through the University of Cambridge data repository: https://doi.org/10.17863/CAM.128183.

## Notes

Synthetic procedures, ^1^H NMR spectra, material characterization, transient absorption spectroscopy data, time-resolved PL plots, OD_600_ measurements, photostability characterization, comparison to state-of-the-art biohybrid systems and raw data for main figures. (PDF)
