# Supplementary material for: Organic Semiconducting Hydrogel with Integrated Microbes and Enzymes for Selective Solar CO2 Conversion
Source: J Am Chem Soc. 2026 Mar 21;148(15):16019–31. doi: 10.1021/jacs.6c00205 (PMC13107442; doi:10.1021/jacs.6c00205)
Supplement: Supplementary file 1 [file ja6c00205_si_001.pdf]

Supporting Information

Organic Semiconducting Hydrogel with Integrated Microbes and Enzymes for Selective Solar CO_2 Conversion

Glenn Quek,[1] Beverly Qian Ling Low,[1] Soleh Anderlini,[1] Xian Wei Chua,[2,3] Marion I. M. Short,[1] Dongseok Kim,[1] Samuel D. Stranks,[2,3] Erwin Reisner[1*]

[1] *Yusuf Hamied Department of Chemistry, University of Cambridge, Lensfield Road, Cambridge CB2 1EW, United Kingdom.*

**Email:* reisner@ch.cam.ac.uk

[2] *Department of Chemical Engineering and Biotechnology, University of Cambridge, Cambridge CB3 0AS, U.K.*

[3] *Department of Physics, Cavendish Laboratory, University of Cambridge, Cambridge CB3 0HE, U.K.*

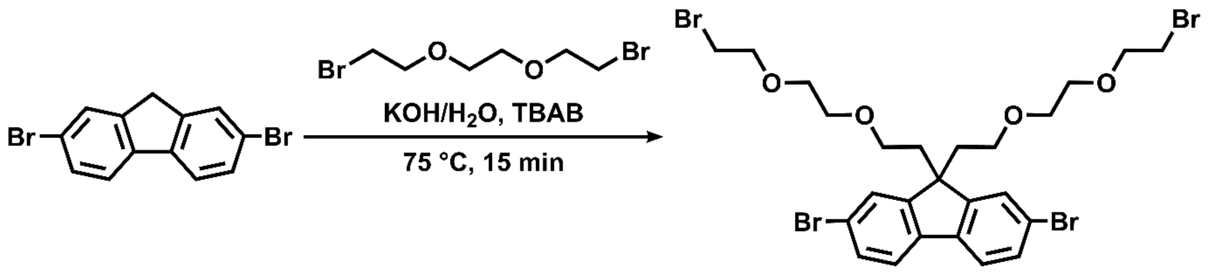

Figure S1. Synthesis of 2,7-Dibromo-9,9-bis(2-(2-(2-methoxyethoxy)ethoxy)ethyl)fluorene.

2,7-Dibromo-9,9-bis(2-(2-(2-methoxyethoxy)ethoxy)ethyl)fluorene was synthesized according to a previous report.[1] 2,7-Dibromofluorene (1.23 g, 5 mmol) was added to a mixture of aqueous potassium hydroxide (100 mL, 50 wt% (13.6 M)), tetrabutylammonium bromide (0.330 g, 1 mmol), and 1,2-bis(2-bromoethoxy)ethane (13.9 g, 50 mmol) at 75 °C. After 15 min, the mixture was cooled to room temperature. After extraction with CH_2Cl_2, the combined organic layers were washed successively with water, aqueous HCl (1 M), water, and brine and then dried over Na_2SO_4. After removal of the solvent and the excess 1,2-bis(2-bromoethoxy)ethane, the residue was purified by silica gel column chromatography using hexane and ethyl acetate as the eluent to afford a white solid (1.80 g, 58% yield). ^{1}H NMR (400 MHz, $CDCl_3$, δ ppm): 7.54−7.46 (m, 6 H), 3.68 (t, 4 H, J = 6.4 Hz), 3.39 (m, 8 H), 3.20 (m, 4 H), 2.80 (t, 4 H, J = 6.4 Hz), 2.34 (t, 4 H, J = 6.4 Hz).

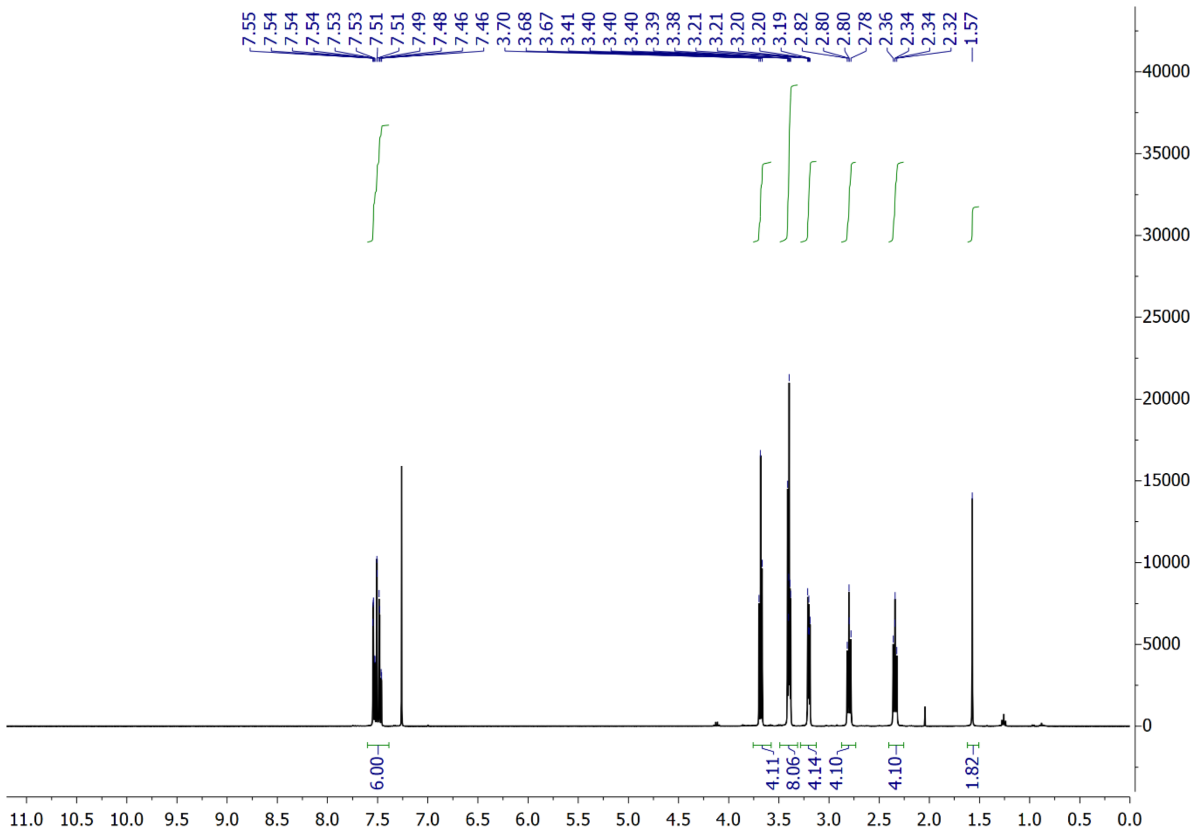

Figure S2. ^{1}H NMR spectrum of 2,7-Dibromo-9,9-bis(2-(2-(2-methoxyethoxy)ethoxy)ethyl)fluorene in deuterated chloroform ($CDCl_3$).

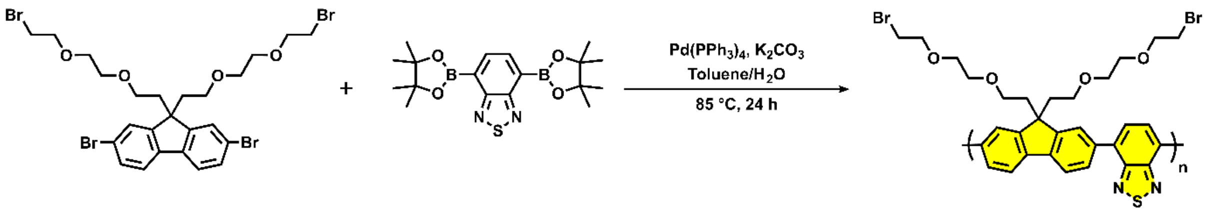

Figure S3. Synthesis of neutral polymer pFBT-Br.

2,7-Dibromo-9,9′-bis(2-(2-(2-bromoethoxy)ethoxy)ethyl)fluorene (1.5 g, 2.1 mmol), 4,7-bis(4,4,5,5-tetramethyl-1,3,2-dioxaborolan-2-yl)benzo[c][1,2,5]thiadiazole (818 mg, 2.1 mmol), $Pd(PPh_3)_4$ (41 mg, 0.035 mmol) and potassium carbonate (7.03 g, 50.9 mmol) were placed in a round-bottom flask. A mixture of water (26 mL) and toluene (45 mL) was added to the flask, and the reaction vessel was degassed. The mixture

was vigorously stirred at 85 °C for 24 h and then precipitated into methanol. The polymer was centrifuged and washed with methanol, water and acetone, and then dried under vacuum for 24 h to afford 1.2 g of yellow solids (80% yield).

^{1}H NMR (400 MHz, $CDCl_3$, δ ppm): 8.15−7.98 (m, 8 H), 3.66 (t, 4 H, *J* = 6.4 Hz), 3.46 (t, 4 H,z *J* = 6.4 Hz), 3.35 (m, 8 H), 3.10 (br, 4 H), 2.61 (br, 4 H). GPC ($CHCl_3$): M_n: 8515 g mol^{-1}, M_W: 35094 g mol^{-1}, PDI: 4.12.

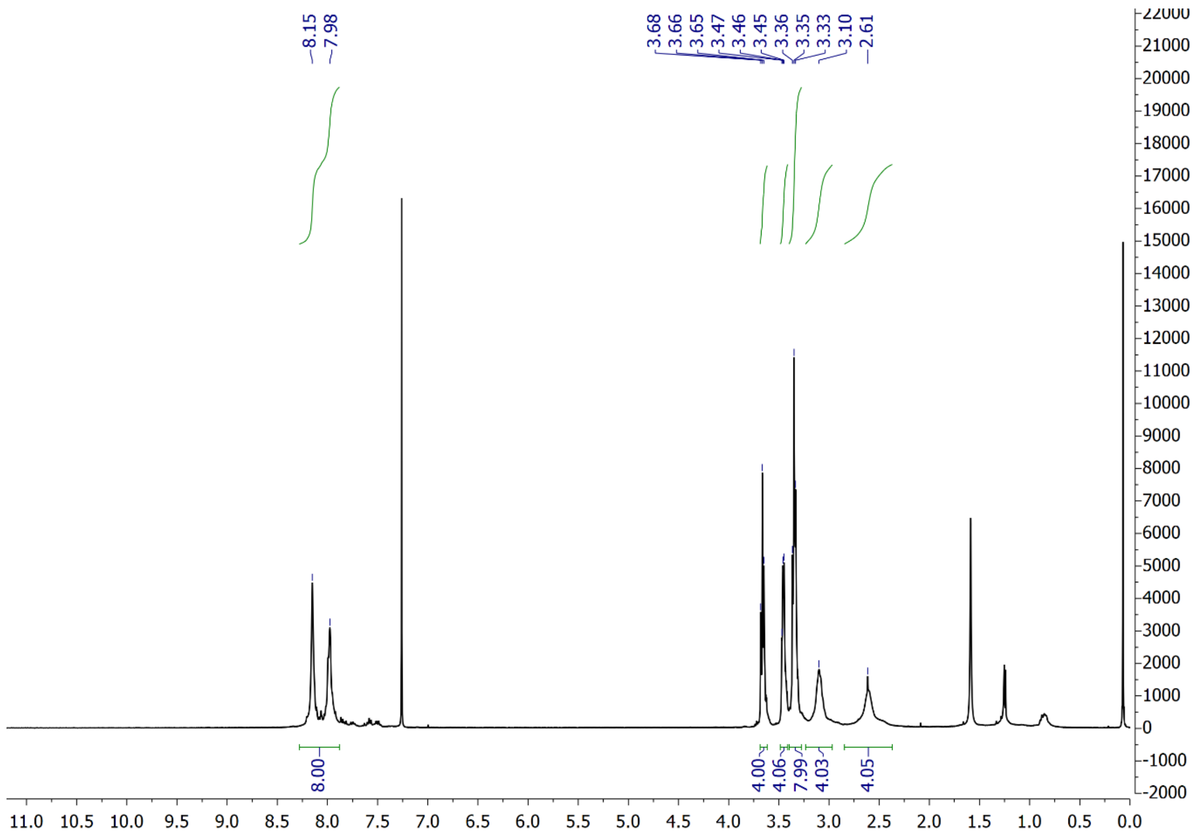

Figure S4. ^{1}H NMR spectrum of pFBT-Br in deuterated chloroform ($CDCl_3$).

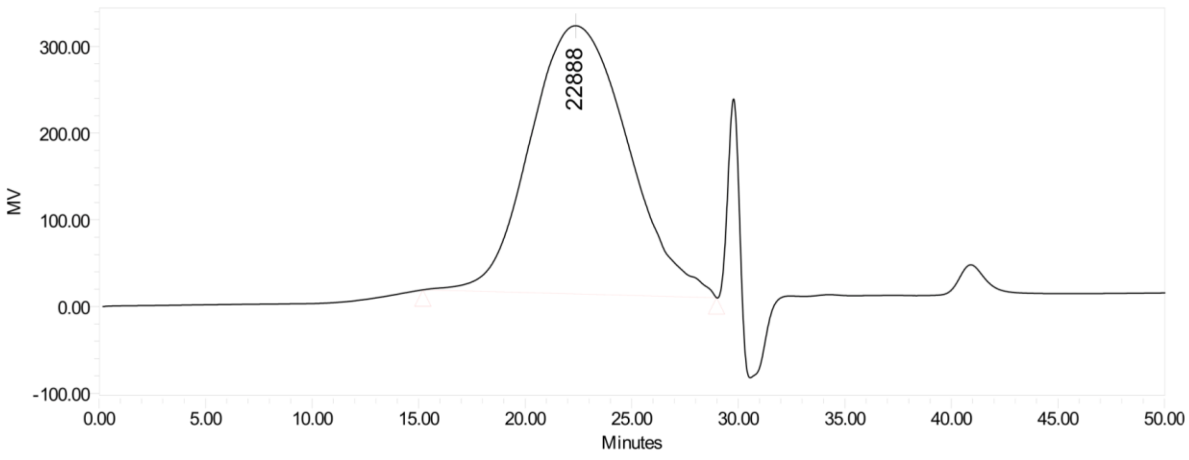

GPC Results

	Retention Time (min)	Mn	Mw	MP	Mz	Mz+1	Mv	Polydispersity
1	22.371	8515	35094	22888	87348	151970		4.121389

Figure S5. Gel permeation chromatography (GPC) plot of pFBT-Br.

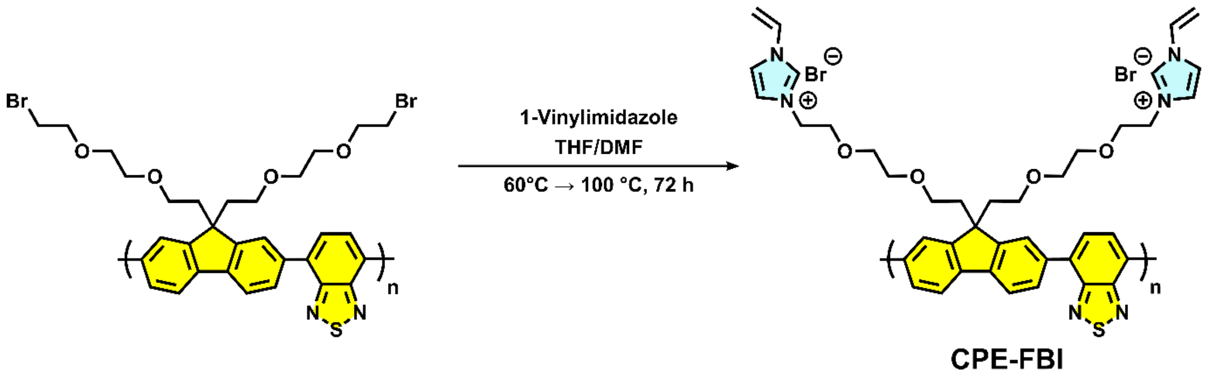

Figure S6. Synthesis of **CPE-FBI**.

pFBT-Br (220 mg) was dissolved in dry THF (4 mL) under N_2 atmosphere, then dry DMF (8 mL), 1-vinylimidazole (10 g), and 2,6-di-tert-butyl-4-methylphenol (0.5 g) were added under stirring. The reaction mixture was heated under dark at 60 °C, 80 °C and 100 °C, each for 24 h, then precipitated into THF. The polymer was centrifuged and washed with THF three times, and then dried under vacuum for 24 h to afford 232 mg of orange solids (84% yield).

^1H NMR (400 MHz, $(CD_3)_2SO$, δ ppm): 9.43 (br, 2 H), 8.34−8.17 (br, 8 H), 7.81 (br, 4 H), 7.28 (br, 2 H), 5.91 (br, 2 H), 5.36 (br, 2 H), 4.29 (br, 4 H), 3.68 (br, 4 H), 3.53−3.36 (br, 8 H), 3.24 (br, 4 H), 2.98 (br, 4 H).

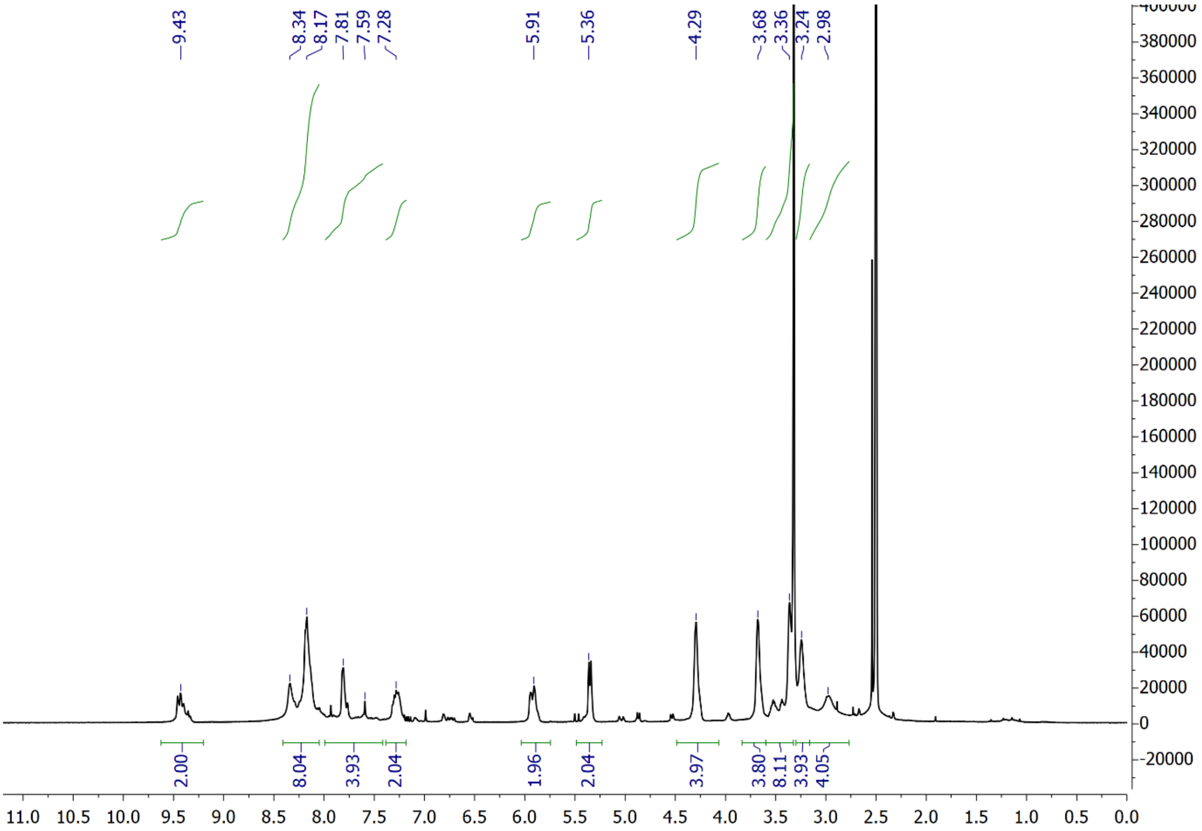

Figure S7. ^1H NMR spectrum of **CPE-FBI** in deuterated DMSO ($(CD_3)_2SO$).

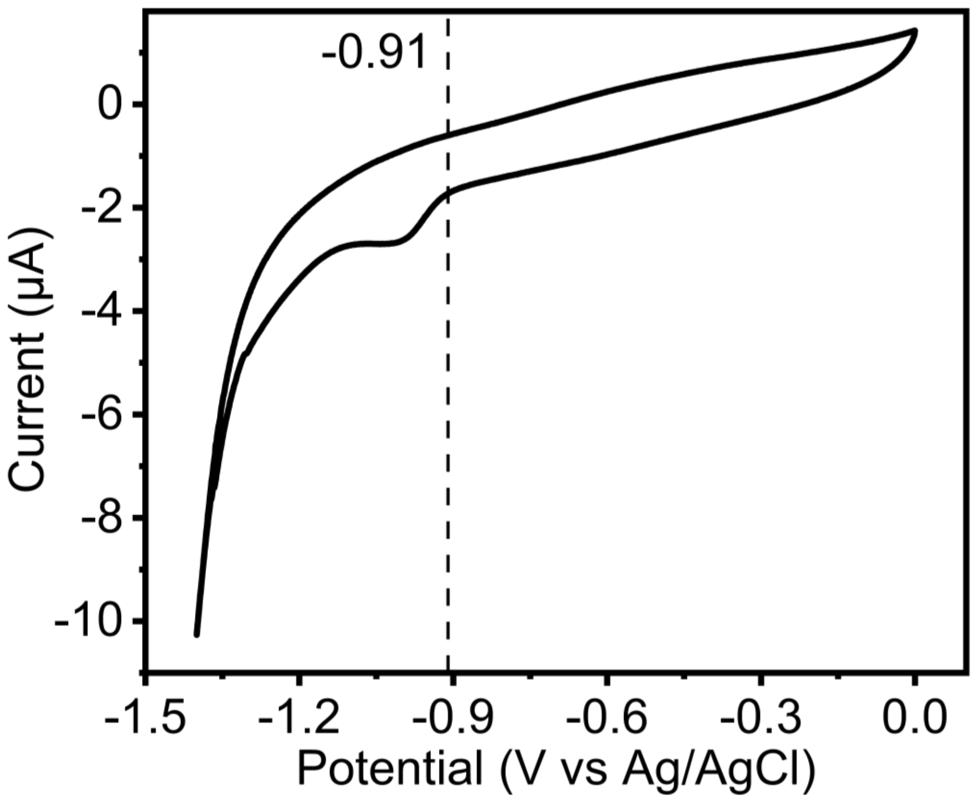

Figure S8. Cyclic voltammetry trace of **CPE-FBI** (0.2 mg mL^{-1}) in H_2O with 100 mM tetrabutylammonium bromide supporting electrolyte. Dashed line indicates the reduction onset potential.

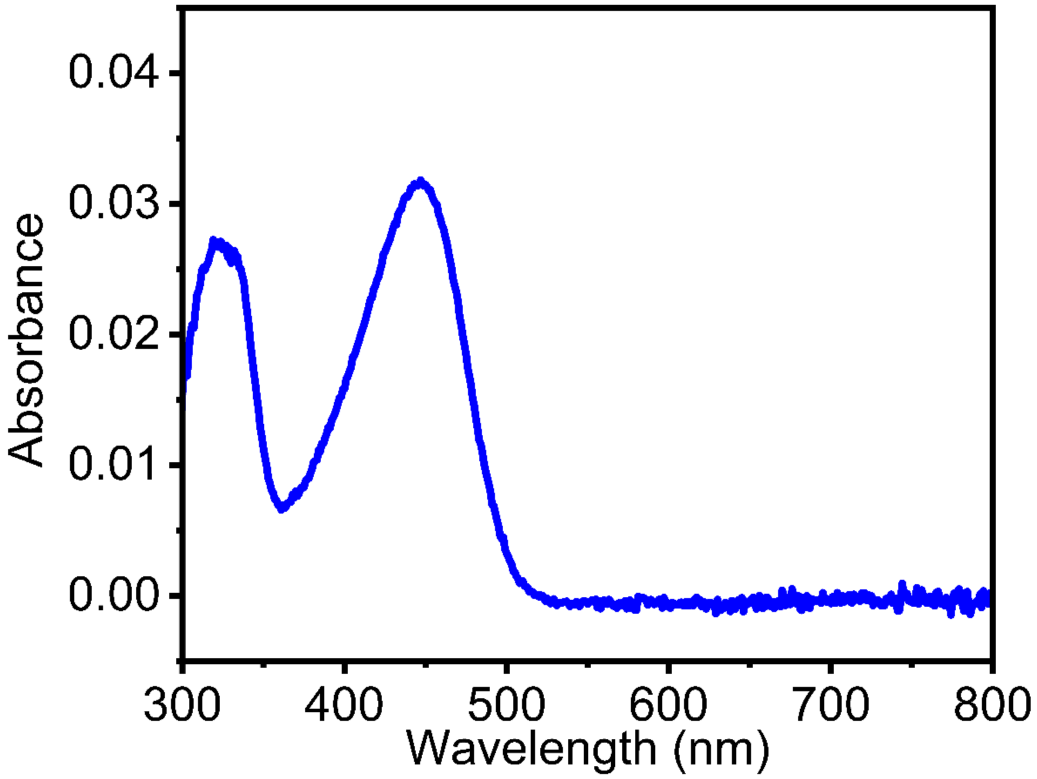

Figure S9. Quantitative determination of **CPE-FBI** covalent crosslinking efficiency. (a) UV-Vis absorbance spectrum of the aqueous supernatant after 72 hours of hydrogel immersion. Based on the absorbance of the supernatant at 450 nm (0.032), the mass of leached **CPE-FBI** was determined to be <1%, corresponding to a covalent crosslinking efficiency of ~99%.

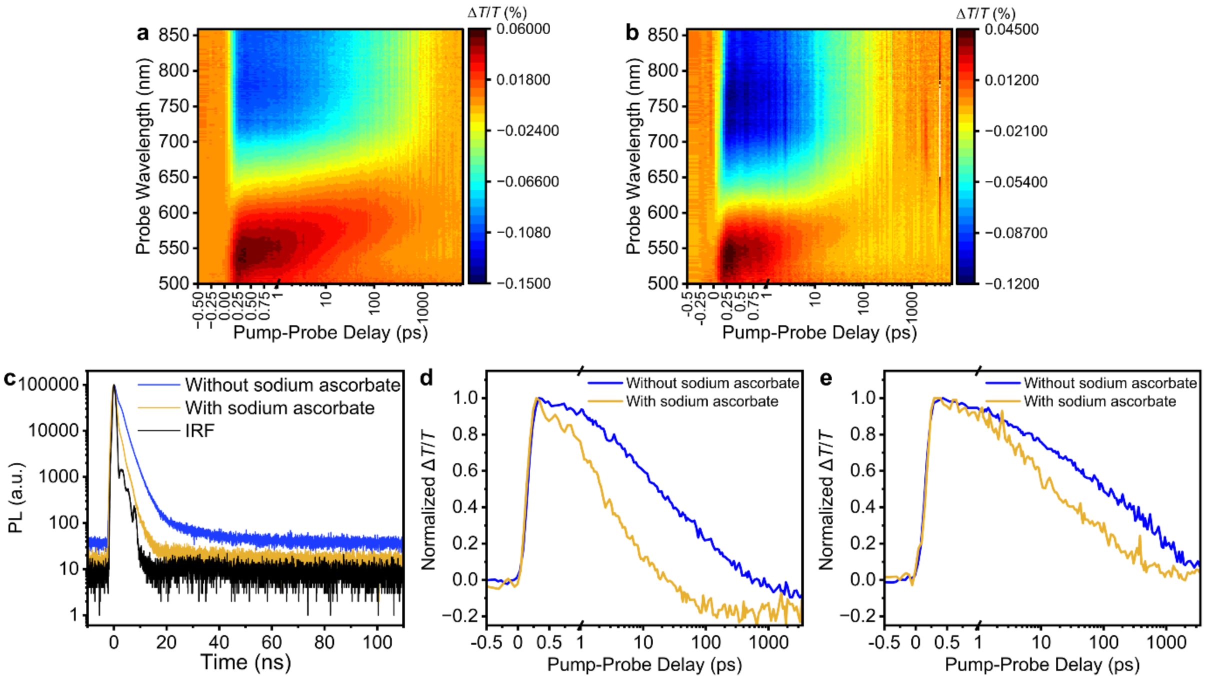

Figure S10. Reductive quenching of photoexcited **CPE-FBI** hydrogels by sodium ascorbate. Transient absorption maps for **CPE-FBI** hydrogels (a) without and (b) with sodium ascorbate (excited at 450 nm with fluence 6.9 μJ cm^{-2}). (c) Time-resolved photoluminescence decays of **CPE-FBI** hydrogels, excited at 450 nm and with the detection wavelength at the PL maxima of 580 nm. Instrument response function is shown in black. The laser operated at a repetition rate of 5 MHz, with an average power of 0.81 mW, corresponding to an energy of 162 pJ per pulse. PL lifetime is reduced from 2.18 ns to 0.98 ns with the addition of sodium ascorbate. Normalized transient absorption kinetics at the (d) positive Δ*T*/*T* feature (550 to 570 nm) and (e) negative ΔT/T feature (760 to 780 nm) (excited at 450 nm with fluence 6.9 μJ cm^{-2}).

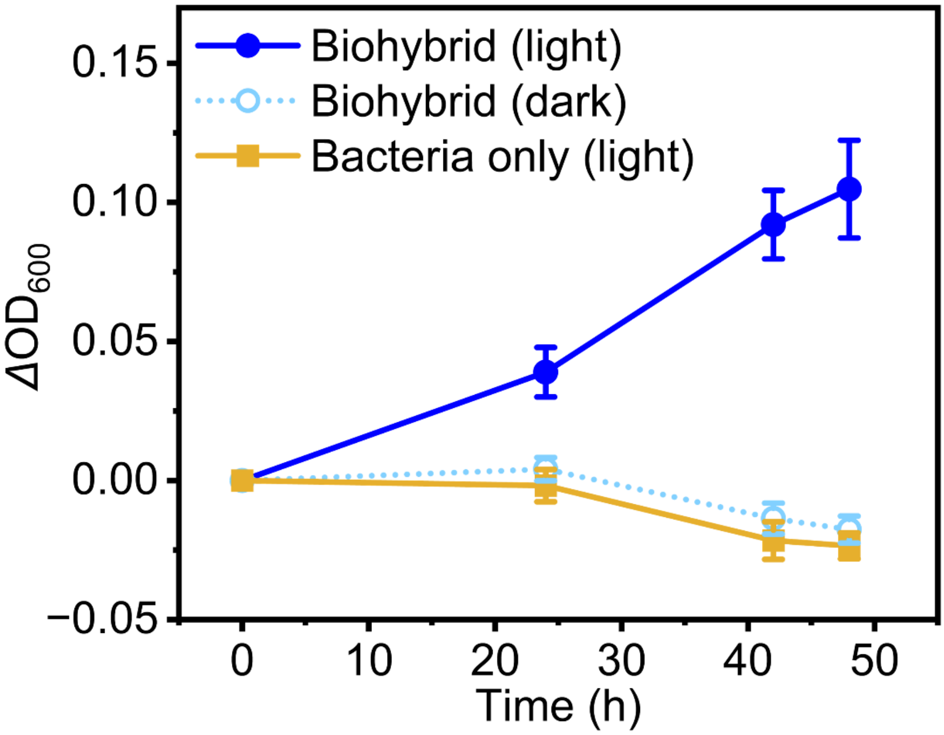

Figure S11. Changes in OD_{600} of *C. ljungdahlii* over 48 hours of reaction for the **CPE-FBI**/*C. ljungdahlii* biohybrid hydrogel in the light and dark, and illuminated control of bacteria only.

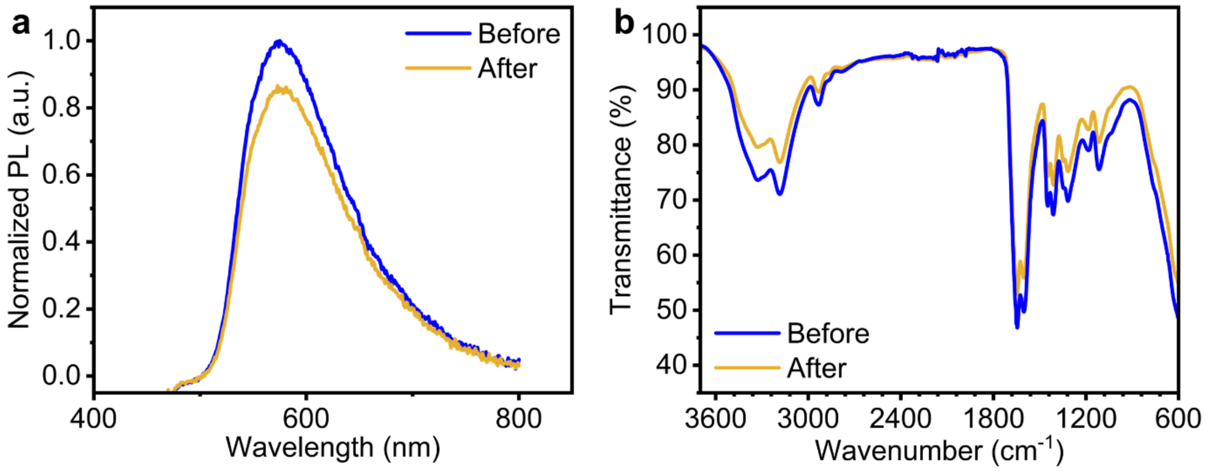

Figure S12. Structural and photophysical stability of the **CPE-FBI** biohybrid hydrogel. (a) PL spectra ($\lambda_{excitation}$ = 450 nm) of the microbial biohybrid hydrogel before and after 48 h of continuous photocatalytic operation. The negligible shift in peak position and intensity confirms the photostability of **CPE-FBI**. (b) FTIR spectra of the microbial biohybrid hydrogel before and after 48 h of continuous photocatalytic operation, showing the preservation of characteristic vibrational modes.

Table S1. Photocatalytic H_2 production by abiotic hydrogels after 24 hours of illumination with varying Pt co-catalyst weight loading relative to that of **CPE-FBI**.

Pt Loading (wt %)	H_2 (μmol g^{-1} h^{-1})
0	n.d.
1.25	207.8 ± 1.5
2.5	433.9 ± 4.2
5	203.5 ± 4.3

Table S2. Photocatalytic product yields from microbial biohybrid hydrogels after 48 hours of illumination: H_2 accumulated in the headspace and acetate produced in the solution.

Sample	Acetate (μmol)		H_2 (μmol)	
	Actual	Electron Equivalents	Actual	Electron Equivalents
**CPE-FBI** only	n.d.	n.d.	13.3 ± 2.4	26.7 ± 4.8
Bacteria only	0.15 ± 0.06	1.2 ± 0.2	n.d.	n.d.
**CPE-FBI**/Bacteria Biohybrid	0.9 ± 0.1	7.1 ± 1.0	5.8 ± 0.4	11.6 ± 0.9

Table S3. Photocatalytic acetate production after 48 hours in deletional control experiments for microbial biohybrid hydrogels.

Components						Acetate (mM)
**CPE-FBI**	Bacteria	Ascorbate	Pt	CO_2	Light	
✓	✓	✓	✓	✓	✓	0.45 ± 0.06
x	✓	✓	✓	✓	✓	0.08 ± 0.01
✓	x	✓	✓	✓	✓	n.d.
✓	✓	x	✓	✓	✓	0.06 ± 0.03
✓	✓	✓	x	✓	✓	0.07 ± 0.01
✓	✓	✓	✓	x	✓	0.08 ± 0.02
✓	✓	✓	✓	✓	x	0.09 ± 0.03

Table S4. Comparison of photocatalytic microbial biohybrid systems for CO_2-to-acetate conversion.

Semiconductor	Microbe	Form Factor	Light Source	Electron Donor	Reaction Time (h)	Bacterial Loading (OD_{600})	Temperature (°C)	Biohybrid Fabrication	Acetate Yield (mM day^{-1})
**CPE-FBI (This work)**	***C. ljungdahlii***	**Hydrogel**	**Xenon Lamp (AM 1.5G, 100 mW cm^{-2}, > 400 nm)**	**Sodium Ascorbate**	**48**	**0.15**	**37**	**Self-assembly**	**~0.45**
CdS[2]	*M. thermoacetica*	Nanoparticle	Xenon Lamp (AM 1.5G, 2 mW cm^{-2})	Cysteine	60	0.16	55	Biomineralization	~0.5
PDI/PFP[3]	*M. thermoacetica*	Nanoparticle /Homogenous	Xenon Lamp (5 mW cm^{-2}, > 420 nm)	Cysteine	72	0.2	52	Self-assembly	~0.18
AuNCs[4]	*M. thermoacetica*	Nanocluster	Xenon Lamp (AM 1.5G, 2 mW cm^{-2})	Cysteine	84	0.28	55	Intracellular uptake	~0.4
Cr_2O_3/Ru-$SrTiO_3$:La,Rh|ITO|RuO_2-$BiVO_4$:Mo[5]	*S. ovata*	Sheet	Xenon Lamp (AM 1.5G, 100 mW cm^{-2})	Water	15	0.6	30	Self-assembly	~14.4
$Cd_{0.8}Zn_{0.2}S$[6]	*S. ovata*	Nanoparticle	Violet LEDs (450 ± 5 nm, 2 mW cm^{-2})	Cysteine	216	-	30	Biomineralization	~3.9
ITO/ZnO/P3HT:PCBM/MoO_3[7]	*S. ovata*	Sheet	White LEDs (10 mW cm^{-2})	Cysteine	168	0.32	34	Self-assembly	~14

Table S5. Photocatalytic formate production by enzyme biohybrid hydrogels with and without MV^{2+} (1mM) after 48 h of reaction.

	Formate (mM)	TON_{FDH} (10^3)
Without MV^{2+}	1.16 ± 0.23	25.7 ± 5.2
With MV^{2+}	1.64 ± 0.20	36.4 ± 4.4

Table S6. Photocatalytic formate production by enzyme biohybrid hydrogels over 2 h illumination cycles, with solution exchange between each cycle.

Cycle	Formate (mM)	TON_{FDH} (10^3)
1st	0.21 ± 0.04	4.7 ± 0.9
2nd	0.19 ± 0.05	4.2 ± 1.1
3rd	0.20 ± 0.04	4.4 ± 0.9

Table S7. Comparison of photocatalytic enzymatic (FDH) biohybrids for CO_2-to-formate conversion.

Semiconductor	Enzyme	Form Factor	Light Source	Electron Donor	Reaction Time (h)	Mechanism	Binding Material/ Functional Group	TOF (h^{-1})
**CPE-FBI (This work)**	***Nv*HFdhAB**	**Hydrogel**	**Xenon Lamp (AM 1.5G, 100 mW cm^{-2}, > 400 nm)**	**Sodium Ascorbate**	**48**	**DET**	**Imidazolium**	**2.0×10^3 (5h)**
RuC_{17}[8]	*Nv*HFdhAB	Micelle	Xenon Lamp (AM 1.5G, 100 mW cm^{-2})	Sodium Ascorbate	24	DET	Ru^{2+}	3.3×10^2 (24h)
$SrTiO_3$:La,Rh|$[Co(bpy)_3]^{3+/2+}$|$BiVO_4$:Mo|RuO_2[9]	*Nv*HFdhAB	Nanoparticle	Xenon Lamp (AM 1.5G, 100 mW cm^{-2})	Water	10	DET	$SrTiO_3$	1.3×10^3 (10h)
CN_X–ITO[10]	*Nv*HFdhAB	Sheet	Xenon Lamp (AM 1.5G, 100 mW cm^{-2})	4-MBA	10	DET	ITO	5.2×10^3 (10h)
a-CD-$NHMe_2^+$[11]	*Nv*HFdhAB	Nanoparticle	Xenon Lamp (AM 1.5G, 100 mW cm^{-2})	DTT	48	DET	Tertiary amine	3.5×10^3 (6h)
RuP|TiO_2[12]	*Nv*HFdhAB	Nanoparticle	Xenon Lamp (AM 1.5G, 100 mW cm^{-2}, > 420 nm)	TEOA	24	DET	TiO_2	3.9×10^4 (6h)
DPP|TiO_2[12]	*Nv*HFdhAB	Nanoparticle	Xenon Lamp (AM 1.5G, 100 mW cm^{-2}, > 420 nm)	TEOA	24	DET	TiO_2	1.7×10^4 (6h)
MOF|Rh complex[13]	CbFDH	Suspension	Halogen Lamp (400-700 nm, 250 W cm^{-2})	TEOA	24	MET (NADH)	MOF	865 (24h)
Graphene-MAQSP|Rh complex[14]	CbFDH	Suspension	Xenon Lamp (450 W, > 420 nm)	TEOA	2	MET (NADH)	-	1.69 (2h)
ZnTMPyP[15]	SceFDH	Homogenous	Tungsten Lamp (200 W, > 390 nm)	TEOA	3	MET (MV)	-	0.10 (3h)
Mg chlorophyll-*a*[16]	SceFDH	Homogenous	Tungsten Lamp (200 J m^{-2} s^{-1}, > 390 nm)	NADPH	3	MET (MV)	-	4.64 (1h)

References

(1) Pu, K.-Y.; Liu, B. Conjugated Polyelectrolytes as Light-Up Macromolecular Probes for Heparin Sensing. *Advanced Functional Materials* **2009**, *19* (2), 277-284. DOI: https://doi.org/10.1002/adfm.200800960.

(2) Sakimoto, K. K.; Wong, A. B.; Yang, P. Self-photosensitization of nonphotosynthetic bacteria for solar-to-chemical production. *Science* **2016**, *351* (6268), 74-77. DOI: doi:10.1126/science.aad3317.

(3) Gai, P.; Yu, W.; Zhao, H.; Qi, R.; Li, F.; Liu, L.; Lv, F.; Wang, S. Solar-Powered Organic Semiconductor–Bacteria Biohybrids for CO_2 Reduction into Acetic Acid. *Angewandte Chemie International Edition* **2020**, *59* (18), 7224-7229. DOI: https://doi.org/10.1002/anie.202001047.

(4) Zhang, H.; Liu, H.; Tian, Z.; Lu, D.; Yu, Y.; Cestellos-Blanco, S.; Sakimoto, K. K.; Yang, P. Bacteria photosensitized by intracellular gold nanoclusters for solar fuel production. *Nature Nanotechnology* **2018**, *13* (10), 900-905. DOI: 10.1038/s41565-018-0267-z.

(5) Wang, Q.; Kalathil, S.; Pornrungroj, C.; Sahm, C. D.; Reisner, E. Bacteria–photocatalyst sheet for sustainable carbon dioxide utilization. *Nature Catalysis* **2022**, *5* (7), 633-641. DOI: 10.1038/s41929-022-00817-z.

(6) Zhang, K.; Li, R.; Chen, J.; Chai, L.; Lin, Z.; Zou, L.; Shi, Y. Biohybrids of twinning $Cd_{0.8}Zn_{0.2}S$ nanoparticles and Sporomusa ovata for efficient solar-driven reduction of CO_2 to acetate. *Applied Catalysis B: Environmental* **2024**, *342*, 123375. DOI: https://doi.org/10.1016/j.apcatb.2023.123375.

(7) Wen, N.; Jiang, Q.; Liu, D. Polymer semiconductor films and bacteria hybrid artificial bio-leaves. *Science Advances* **2024**, *10* (44), eadp8567. DOI: doi:10.1126/sciadv.adp8567.

(8) Liu, Y.; Rodríguez-Jiménez, S.; Song, H.; Pannwitz, A.; Kim, D.; Coito, A. M.; Manuel, R. R.; Webb, S.; Su, L.; Bonke, S. A.; et al. Bio-Inspired Self-Assembly of Enzyme-Micelle Systems for Semi-Artificial Photosynthesis. *Angewandte Chemie International Edition* **2025**, *64* (18), e202424222. DOI: https://doi.org/10.1002/anie.202424222.

(9) Liu, Y.; Bin Mohamad Annuar, A.; Rodríguez-Jiménez, S.; Yeung, C. W. S.; Wang, Q.; Coito, A. M.; Manuel, R. R.; Pereira, I. A. C.; Reisner, E. Solar Fuel Synthesis Using

a Semiartificial Colloidal Z-Scheme. *Journal of the American Chemical Society* **2024**, *146* (43), 29865-29876. DOI: 10.1021/jacs.4c11827.

(10) Rahaman, M.; Pulignani, C.; Miller, M.; Bhattacharjee, S.; Bin Mohamad Annuar, A.; Manuel, R. R.; Pereira, I. A. C.; Reisner, E. Solar-Driven Paired CO2 Reduction–Alcohol Oxidation Using Semiartificial Suspension, Photocatalyst Sheet, and Photoelectrochemical Devices. *Journal of the American Chemical Society* **2025**, *147* (10), 8168-8177. DOI: 10.1021/jacs.4c10519.

(11) Badiani, V. M.; Casadevall, C.; Miller, M.; Cobb, S. J.; Manuel, R. R.; Pereira, I. A. C.; Reisner, E. Engineering Electro- and Photocatalytic Carbon Materials for CO_2 Reduction by Formate Dehydrogenase. *Journal of the American Chemical Society* **2022**, *144* (31), 14207-14216. DOI: 10.1021/jacs.2c04529.

(12) Miller, M.; Robinson, W. E.; Oliveira, A. R.; Heidary, N.; Kornienko, N.; Warnan, J.; Pereira, I. A. C.; Reisner, E. Interfacing Formate Dehydrogenase with Metal Oxides for the Reversible Electrocatalysis and Solar-Driven Reduction of Carbon Dioxide. *Angewandte Chemie International Edition* **2019**, *58* (14), 4601-4605. DOI: https://doi.org/10.1002/anie.201814419.

(13) Chen, Y.; Li, P.; Zhou, J.; Buru, C. T.; Đorđević, L.; Li, P.; Zhang, X.; Cetin, M. M.; Stoddart, J. F.; Stupp, S. I.; et al. Integration of Enzymes and Photosensitizers in a Hierarchical Mesoporous Metal–Organic Framework for Light-Driven CO2 Reduction. *Journal of the American Chemical Society* **2020**, *142* (4), 1768-1773. DOI: 10.1021/jacs.9b12828.

(14) Yadav, R. K.; Baeg, J.-O.; Oh, G. H.; Park, N.-J.; Kong, K.-j.; Kim, J.; Hwang, D. W.; Biswas, S. K. A Photocatalyst–Enzyme Coupled Artificial Photosynthesis System for Solar Energy in Production of Formic Acid from CO_2. *Journal of the American Chemical Society* **2012**, *134* (28), 11455-11461. DOI: 10.1021/ja3009902.

(15) Miyatani, R.; Amao, Y. Photochemical synthesis of formic acid from CO2 with formate dehydrogenase and water-soluble zinc porphyrin. *Journal of Molecular Catalysis B: Enzymatic* **2004**, *27* (2), 121-125. DOI: https://doi.org/10.1016/j.molcatb.2003.11.003.

(16) Tsujisho, I.; Toyoda, M.; Amao, Y. Photochemical and enzymatic synthesis of formic acid from CO_2 with chlorophyll and dehydrogenase system. *Catalysis Communications* **2006**, *7* (3), 173-176. DOI: https://doi.org/10.1016/j.catcom.2005.10.005.
